# Genomic legacy of the African cheetah, *Acinonyx jubatus*

**DOI:** 10.1186/s13059-015-0837-4

**Published:** 2015-12-10

**Authors:** Pavel Dobrynin, Shiping Liu, Gaik Tamazian, Zijun Xiong, Andrey A. Yurchenko, Ksenia Krasheninnikova, Sergey Kliver, Anne Schmidt-Küntzel, Klaus-Peter Koepfli, Warren Johnson, Lukas F.K. Kuderna, Raquel García-Pérez, Marc de Manuel, Ricardo Godinez, Aleksey Komissarov, Alexey Makunin, Vladimir Brukhin, Weilin Qiu, Long Zhou, Fang Li, Jian Yi, Carlos Driscoll, Agostinho Antunes, Taras K. Oleksyk, Eduardo Eizirik, Polina Perelman, Melody Roelke, David Wildt, Mark Diekhans, Tomas Marques-Bonet, Laurie Marker, Jong Bhak, Jun Wang, Guojie Zhang, Stephen J. O’Brien

**Affiliations:** Theodosius Dobzhansky Center for Genome Bioinformatics, Saint Petersburg State University, 41A Sredniy Avenue, St. Petersburg, 199004 Russia; National Genbank, BGI-Shenzhen, Shenzhen, 518083 China; National Zoological Park, Smithsonian Conservation Biology Institute, Washington DC, 20007 USA; Institut de Biologia Evolutiva (CSIC/UPF), Dr. Aiguader, 88, Barcelona, 08003 Spain; Department of Organismic and Evolutionary Biology and Museum of Comparative Zoology, Harvard University, Cambridge, 02138 Massachusetts USA; Laboratory of Neurogenetics, NIAAA, 5625 Fishers Lane, Rockville, 20852 Maryland USA; CIIMAR/CIMAR, Interdisciplinary Centre of Marine and Environmental Research, University of Porto, Rua dos Bragas, 177, Porto, 4050-123 Portugal; Department of Biology, Faculty of Sciences, University of Porto, Rua do Campo Alegre, Porto, 4169-007 Portugal; Biology Department, University of Puerto-Rico at Mayaguez, Mayaguez, Puerto Rico; PUCRS, Faculdade de Biociencias, Laboratorio de Biología Genómica e Molecular, Porto Alegre, 90619-900 Brazil; Institute of Molecular and Cellular Biology of the Russian Academy of Sciences, Novosibirsk, 630090 Russia; Novosibirsk State University, Novosibirsk, 630090 Russia; Laboratory of Animal Sciences Progras, Leídos Biomedical Research Inc., Frederick National Laboratory, Frederick, 21702 Maryland USA; Center for Biomolecular Science and Engineering, University of California, Santa-Cruz, USA; Life Technologies Conservation Genetics Laboratory, Cheetah Conservation Fund, Otjiwarongo, Otjiwarongo, 9000 Namibia; Cheetah Conservation Fund, Otjiwarongo, Otjiwarongo, 9000 Namibia; Biomedical Engineering Department, UNIST, Ulsan National Institute of Science and Technology, Ulsan, Korea; BGI-Shenzhen, Shenzhen, 518083 China; Department of Biology, University of Copenhagen, Ole Maaløes Vej 5, Copenhagen, 2200 Denmark; Princess Al Jawhara Center of Excellence in the Research of Hereditary Disorders, King Abdulaziz University, Jeddah, 21589 Saudi Arabia; Macau University of Science and Technology, Taipa, 999078 Macau China; Oceanographic Center, Nova Southeastern University Ft Lauderdale, 8000 N. Ocean Drive, Ft Lauderdale, 33004 Florida USA; Instituto Catalana de Recerca i Estidus Avancats (ICREA), Barcelona, Spain; Centro Nacional de Analisis Genomics (CNAG), Baldiri Reixach 4, Barcelona, 08013 Spain; State Key Laboratory of Biocontrol, School of Life Sciences, Sun Yat-sen University, Guangzhou, 510006 PR China; Centre for Social Evolution, Department of Biology, University of Copenhagen, Universitetsparken 15, Copenhagen, DK-2100 Denmark

**Keywords:** Genetic diversity, Conservation biology, Population biology

## Abstract

**Background:**

Patterns of genetic and genomic variance are informative in inferring population history for human, model species and endangered populations.

**Results:**

Here the genome sequence of wild-born African cheetahs reveals extreme genomic depletion in SNV incidence, SNV density, SNVs of coding genes, MHC class I and II genes, and mitochondrial DNA SNVs. Cheetah genomes are on average 95 % homozygous compared to the genomes of the outbred domestic cat (24.08 % homozygous), Virunga Mountain Gorilla (78.12 %), inbred Abyssinian cat (62.63 %), Tasmanian devil, domestic dog and other mammalian species. Demographic estimators impute two ancestral population bottlenecks: one >100,000 years ago coincident with cheetah migrations out of the Americas and into Eurasia and Africa, and a second 11,084–12,589 years ago in Africa coincident with late Pleistocene large mammal extinctions. MHC class I gene loss and dramatic reduction in functional diversity of MHC genes would explain why cheetahs ablate skin graft rejection among unrelated individuals. Significant excess of non-synonymous mutations in *AKAP4* (*p*<0.02), a gene mediating spermatozoon development, indicates cheetah fixation of five function-damaging amino acid variants distinct from *AKAP4* homologues of other Felidae or mammals; *AKAP4* dysfunction may cause the cheetah’s extremely high (>80 %) pleiomorphic sperm.

**Conclusions:**

The study provides an unprecedented genomic perspective for the rare cheetah, with potential relevance to the species’ natural history, physiological adaptations and unique reproductive disposition.

**Electronic supplementary material:**

The online version of this article (doi:10.1186/s13059-015-0837-4) contains supplementary material, which is available to authorized users.

## Background

The African cheetah—the world’s fastest land animal—is a paradigm of physical prowess that displays numerous physiological adaptations allowing for magnificent high-speed sprints across the African plains. Cheetahs have elongated legs, slim aerodynamic skulls and enlarged adrenal glands, liver and heart, plus semi-retractable claws that grip the earth like football cleats as they race after prey at >100 km/hour. Cheetahs have captured the imagination of artists, writers, regal potentates and wildlife lovers for centuries. Initially descended from early Pliocene precursors related to American pumas, their fossil record extends across the Americas, Europe and Asia until the late Pleistocene (∼10,000–12,000 years ago) when an abrupt extinction after the last glacial retreat extirpated ∼40 species of large mammals, including cheetahs and pumas from North America [1–5].

Modern cheetahs range across eastern and southern Africa (a small number are in Iran, a relict of the Asiatic cheetah subspecies [6]) and are considered highly endangered by wildlife authorities and governments. As a species, cheetahs show a dramatic reduction in overall genetic variation revealed by multiple genomic markers, including an ability to accept reciprocal skin grafts from unrelated cheetahs [7–9]. Their genetic depletion correlates with elevated juvenile mortality, extreme abnormalities in sperm development, difficulties until recently in achieving sustainable captive breeding, and increased vulnerability to infectious disease outbreaks [10–13]. Cheetahs today remain a conservation icon and a symbol for the cost of genetic impoverishment caused by demographic reduction, close inbreeding and near extinction in small free-ranging natural populations. Genetic loss in modern cheetahs has been debated, validated and researched on multiple levels, and is believed to derive from one or more severe population bottlenecks that occurred over time and space during the Pleistocene epoch [7, 14–18]. That precipitous drop in number and genetic diversity, aggravated by behavioral reinforcement of immense range boundaries, led to the genetically depleted cheetah populations surviving today.

Here we present a detailed annotation and analysis of the assembled whole-genome sequence of African cheetah that affirms the genome-wide reduction of cheetah diversity and identifies gene adaptations that occurred in the cheetah’s evolutionary lineage.

## Results

DNA from a male Namibian cheetah, Chewbaaka, was parsed into seven mate-pair libraries and sequenced to high (75-fold) coverage on Illumina HiSeq2000 and assembled de novo (Additional file 1: Figures S1–S3; Additional file 2: Tables S1, S3–S5). Cheetah genome scaffolds (2332 scaffolds; N50 contig: 28.2 kbp, N50 scaffold: 3.1 Mb) were aligned to the reference *Felis catus 6.2* cat genome assembly (hereafter called *Fca-6.2*) anchored with linkage and radiation hybrid maps [19, 20] as well as to the genomes of the lion (*Panthera leo*), tiger (*P. tigris*) and domestic dog (*Canis familiaris*) using a multiple sequence alignment estimated with the Progressive Cactus software [21]. Features of the cheetah genome were annotated from the alignments including 20,343 protein-coding genes, repeat families (∼39.5 % of the genome) and single nucleotide variants (SNVs) (Table 1; Additional file 2: Tables S6–S11 and S15). Comparative analysis of cat (*Felis catus*), cheetah, lion and tiger genomes using the GRIMM and GRIMM Synteny tools [22] identified 220 breakpoints including 19–121 exchanges among different felids (Additional file 1: Figures S5 and S6; Additional file 2: Tables S13 and S14). The aligned cheetah and cat *Fca-6.2* assemblies with annotated genomic feature details (Table 1) are publicly posted in the GARfield browser (http://garfield.dobzhanskycenter.org) and the hub for the UCSC Genome Browser (http://genome.ucsc.edu).

**Table 1:** Assembly and annotation of the cheetah genome

Number	Feature		Size	Source
	Genome sequence and assembly	7 cheetahs		
1	*A. jubatus raineyii* (Tanzania)	3 cheetahs	75 × reference	Table S2
2	*A. jubatus jubatus* (Namibia)	4 cheetahs	5 × resequencing	Table S2
3	SOAP deNovo assembly			Tables S1, S4
4	Assisted assembly with domestic cat *Fca-6.2*	*Fca-6.2* framework anchors:		Table S5
		a. Radiation Hybrid map	3000 markers	
		b. Linkage map	60,000 SNVs	
5	Estimated genome size (assembly and 17-mer)		2.375–2.395 Gb	Table S3
6	N50 contigs		28.2 kbp	Table S4
7	N50 scaffolds		3.1 Mb	Table S4
8	Average GC content		0.475	Figure S3
	Annotation			
9	Coding genes	20,343 genes	601.2 Mb	Table S10
10	Non-coding RNA 200,045 loci		17 Mb	Table S11
		a. 43,878 microRNA	4.41 Mb	Table S11
		b. 1,605 small nuclear RNA	186 kbp	Table S11
		c. 154,031 transport RNA	12.7 Mb	Table S11
		d. 531 ribosomal RNA	85 kbp	Table S11
11	Single nucleotide variants (SNVs)		1,820,419 loci	Tables S15–S20
12	Repetitive elements	Interspersed repeats	746 Mb	Tables S6, S7
	39.48 % of cheetah genome	Tandem repeats	51.2 Mb	Table S8
		Complex tandem repeats	2.04 Mb	Table S9
		3,126 loci		
		Microsatellites	23.47 Mb	Table S8
		487,898 loci		
13	Genomic rearrangements of cheetah vs domestic cat		93 Mb	Figures S5, S6
				Tables S13, S14
14	Nuclear mitochondrial segments		105.6 kbp	Table S12
15	Positively selected genes		946 genes	Datasheet S5
16	GARfield Genome Browser	http://garfield.dobzhanskycenter.org		

**Fig. 1:**
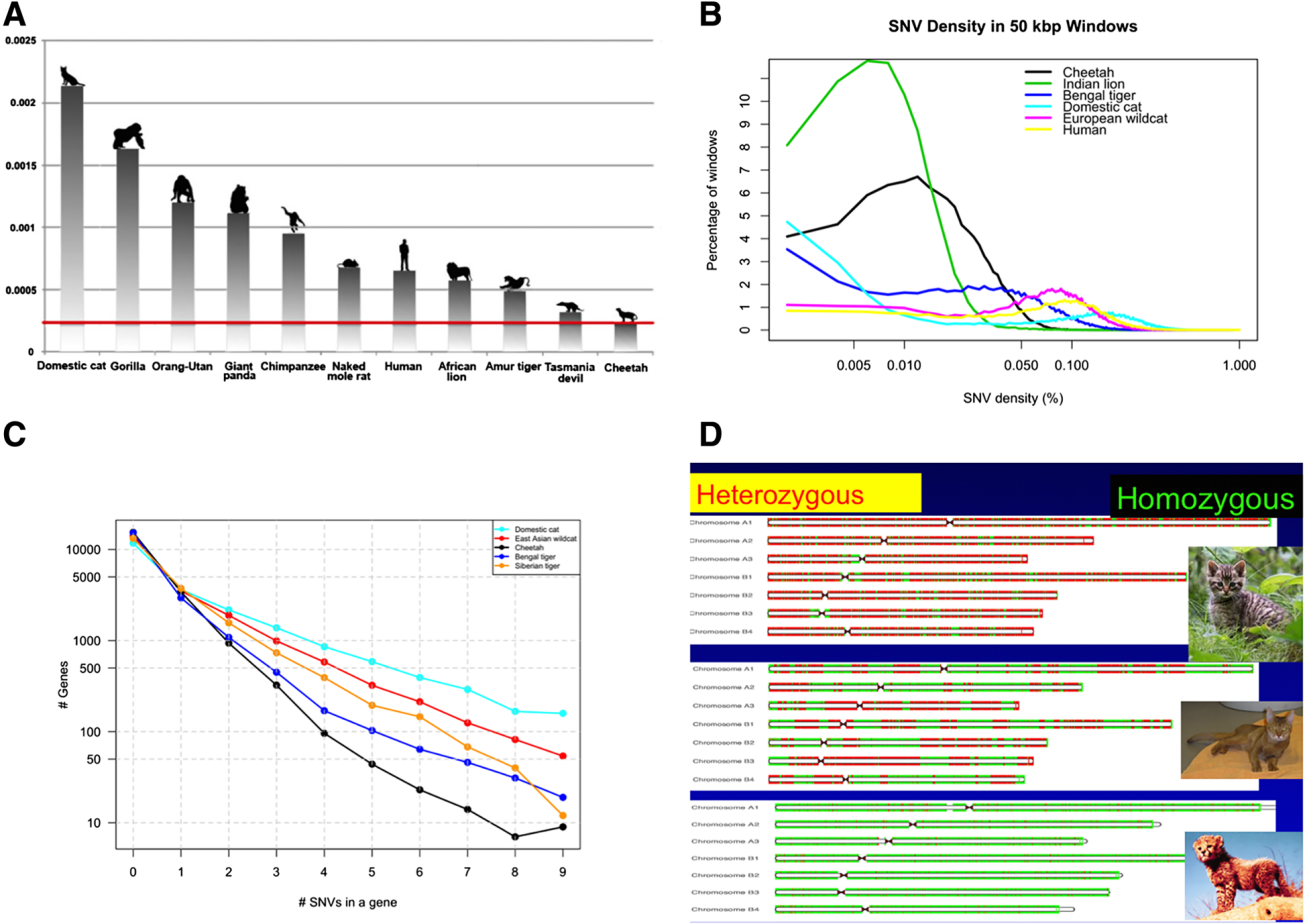
Estimates of genome diversity in the cheetah genome relative to other mammal genomes. **a** SNV rate in mammals. SNV rate for each individual was estimated using all variant positions, with repetitive regions not filtered. **b** SNV density in cheetahs, four other felids and human based upon estimates in 50-kbp sliding windows. Of these, 38,661 fragments had lengths less than the specified window size and thus were excluded from further analysis; most of those fragments are contigs with length less than 500 bp, and thus 46,787 windows of total length 2.337 Gb were built and analyzed. **c** Number of SNVs in protein-coding genes in felid genomes. **d** The cheetah genome is composed of 93 % homozygous stretches. The genome of Boris, an outbred feral domestic cat living in St. Petersburg (*top*) is compared to Cinnamon, a highly inbred Abyssinian cat (*Fca-6.2* reference for domestic cat genome sequence [19, 20], *middle*) and a cheetah (Chewbacca, *bottom*) as described here. Approximately 15,000 regions of 100 Mb across the genome for each species were assessed for SNVs. Regions of high variability (>40 SNVs/100 kbp) are colored *red*; highly homozygous regions (≤40 SNVs/100 kbp) are colored *green*. The first seven chromosome homologues of the genomes of Boris, Cinnamon and Chewbacca are displayed for direct comparison. The median lengths of homozygosity stretches in cheetahs (seven individuals), African lions (five individuals), Siberian and Bengal tigers, and the domestic cat are presented in Additional file 1: Figure S7

**Fig. 2:**
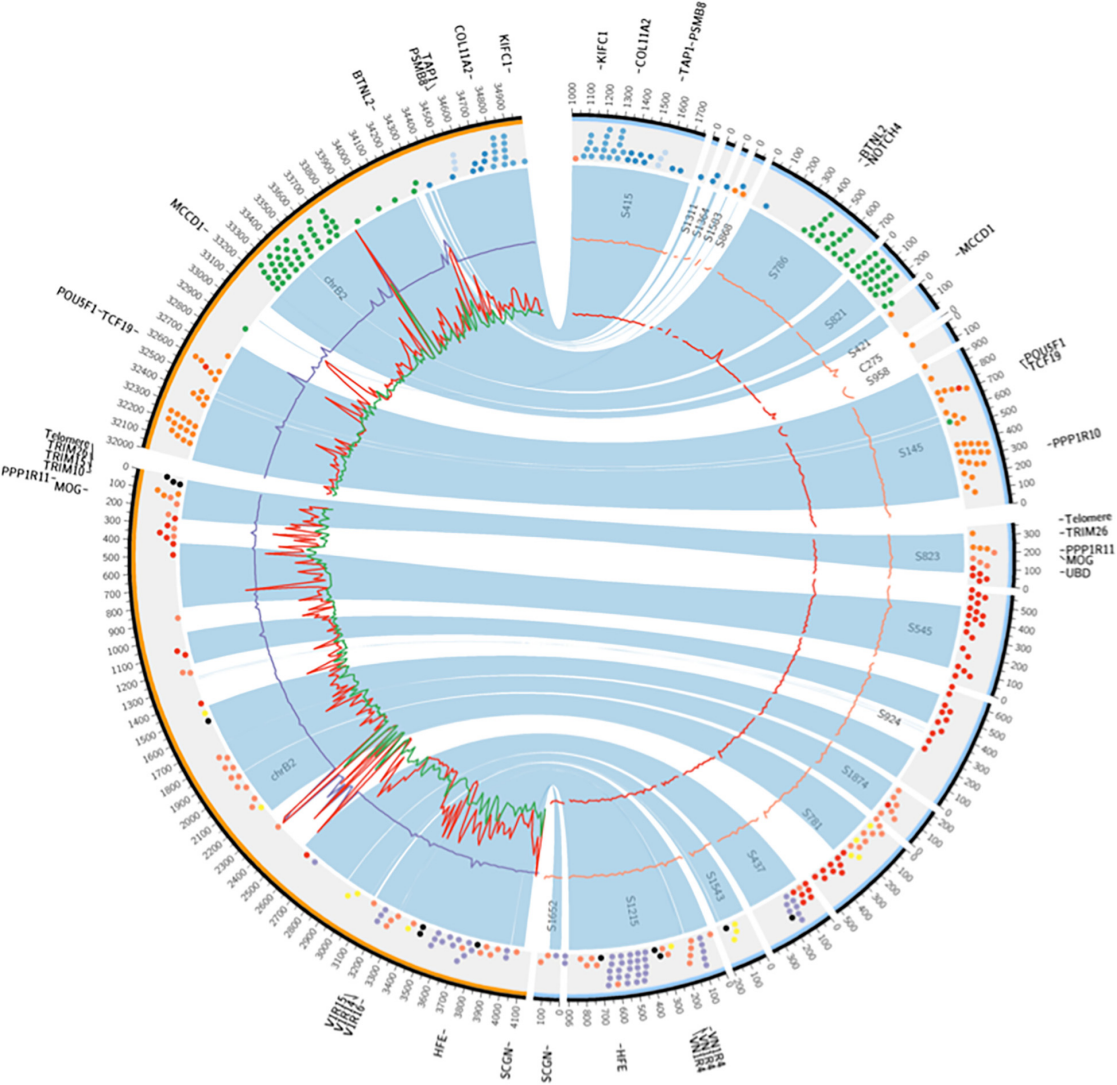
Comparison of MHC region structure between cheetah and domestic cats. *Left side*: Two chromosome B2 segments containing domestic cat MHC genes ordered on BAC libraries [29, 30]. *Right side*: Cheetah scaffolds related to MHC region. Order of scaffolds is based on the results of synteny analysis (*light blue fill*). Individual genes are denoted by *dots* and colored according to their MHC class: *light blue* for extended class II, *blue* for class II, *green* for class III, *orange* for class I, *red* for olfactory receptors and *purple* for histones. Genetic diversity in the MHC region was estimated by calculating SNV counts in non-overlapping 50-kbp windows. These counts are visualized by *colored lines* in the plot; for cats: *green* for wildcat, *red* for Boris and *purple* for Cinnamon; for cheetahs: *red* for Tanzania and *orange* for Namibia

Three additional cheetahs from Tanzania and three from Namibia were sequenced at low coverage (5–6-fold; 500 bp insert size; Additional file 1: Figure S4; Additional file 2: Table S2) and 1,820,419 variable nucleotide sites were identified and compared to SNV variation in other species of Felidae and mammals (Figs. 1 and 2; Additional file 2: Tables S15–S24). We assessed the extent and pattern of genomic diversity using seven different measures, each of which affirmed the remarkable reduction in the cheetah’s genic and genomic variability. First, cheetahs display the lowest overall genome-wide SNV incidence among 11 species including the human, domestic cat, gorilla, lion and Tasmanian devil, and 90 % less than a feral domestic cat (Boris from St. Petersburg; Fig. 1a) [19]. Second, genomes were parsed into 50-kbp windows, which were used to estimate SNV density; in total, 46,787 windows comprised 2.337 Gb or 99.12 % of the total length of the genome. The majority of windows showed 8–15-fold less variation in cheetahs than in the human, domestic cat or wildcat (Fig. 1b). The only sampled species or population with comparable or lower genomic variation than the cheetah was the Gir Forest lions from Asia, a population known to have undergone extreme genetic homogenization in its recent history [23–27].

Third, cheetah coding genes showed dramatic genetic diminution as great as 50-fold (∼98 %) relative to domestic cat or wildcat genome variation (Fig. 1c). The extreme reduction in coding gene variants would explain the initial discovery of the cheetah’s depauperate genetic variation three decades ago with studies using allozymes, cellular protein electrophoretic variants and gene-based restriction fragment length polymorphism (RFLP) [7–9]. Fourth, cheetahs show on average 10–15-fold longer homozygous stretches relative to the feral domestic cat genome; on average 93 % of each cheetah’s genome was homozygous (Fig. 1d; Additional file 1: Figure S8). Fifth, cheetah genomes show far less heterozygous SNV sites, 0.019–0.021 %, reduced to 50–61 % of the incidence in tigers, 30 % of humans and 15 % of domestic cats [19, 28] (Additional file 2: Tables S20 and S21). Sixth, complete mitochondrial genomes of cheetah similarly show on average 90 % reduction in SNVs relative to other species (Additional file 2: Table S25).

Seventh, we also investigated in detail the cheetahs’ major histocompatibility complex (MHC), a cluster of ∼280 immune-related genes, given their functional role and the remarkable observation that cheetahs accepted reciprocal skin allografts from unrelated individuals as if they were immunological “self” [9]. An assisted assembly of 20 cheetah MHC sequence scaffolds on the domestic cat BAC library MHC assembly (total size 8.3 Mb) [29, 30] resolved 278 genes from extended class II, class II, class I and extended class I regions. Although most regions were well covered, complete homologues of certain class I MHC genes (*FLA-I F*, *H* and *M*) were not detected (Additional file 1: Figures S9 and S10; Additional file 2: Table S26). When we compared the structural organization and gene order of the MHC with other species, the cheetah and domestic cat were highly similar, but different from the dog and human. Cheetah and cat MHCs include three functional vomeronasal receptor genes (important for pheromone recognition [31]) in the extended class I region (these genes are absent in the human, nonhuman primates and dog). The cat and cheetah also displayed expansion of certain olfactory receptor genes (0.9 Mb and 30 genes) within the extended class I region [20]. We compared the number of detected SNV variants (synonymous and non-synonymous) in the MHC immune genes from the cheetah (from Namibia and Tanzania), domestic cat, wildcat, human and dog [19, 20, 32]. We found a 95–98 % reduction in both populations of cheetahs and also for Cinnamon (a highly inbred Abyssinian cat who supplied the reference domestic cat genome *Fca-6.2*) [19, 20] relative to abundant SNVs in an outbred domestic cat (Boris), human and dog MHC regions (Fig. 2). The MHC-SNV reductions in the inbred cat and cheetah involve both synonymous and non-synonymous amino acid-altering substitutions. These numerous function-altering variants reflect a history of pathogen-based frequency-dependent selection driving MHC diversity higher across mammals (Additional file 2: Table S26) [33].

**Fig. 3:**
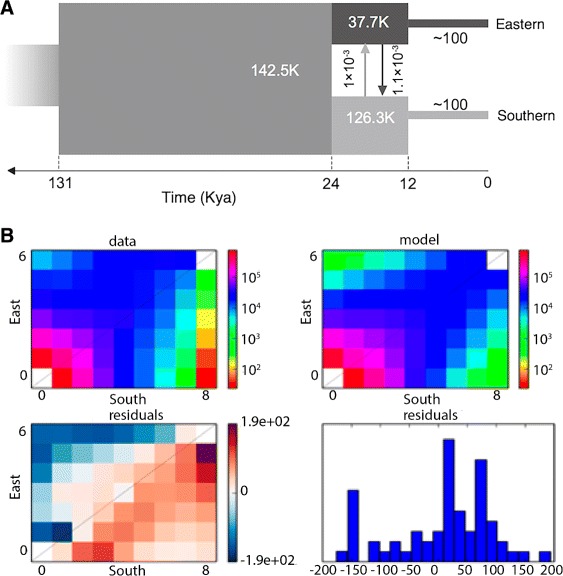
Demographic history analysis of African cheetah. **a** Demographic history of two cheetah populations (southern in Namibia and eastern in Tanzania) based on DaDi analyses. Four distinctive but plausible model scenarios were simulated by the DaDi analysis with the AFS data. Model 4 fits the data best; see “Materials and methods” for our decision algorithm pathway that identified model 4 as best. **b** First and second graphs represent marginal spectra for a pair of populations. The third graph shows residuals between the model and the observed data. *Red* or *blue* residuals indicate that the model predicts too many or too few alleles in a given cell, respectively. The fourth graph shows goodness-of-fit tests based on the likelihood and Pearson’s statistic, with both indicating that our model is a reasonable, though incomplete, description of the data

Patterns of whole-genome sequence variation were used to model and infer the population history of cheetahs from eastern and southern Africa (from Tanzania and Namibia, respectively) using the diffusion approximation to the allele frequency spectrum (AFS) implemented in the DaDi software tool [34]. The DaDi approximation compares the expected allele frequency and the observed AFS over the parameter value space by computing a composite-likelihood score for the best of distinctive but plausible evolutionary scenarios. The scenarios were simulated with the AFS data and the results were used to calculate the likelihoods of best fit for each model (see Fig. 3 legend and “Materials and methods” for the decision algorithm pathway that identified the optimal model).

Model 4 (also denoted by 2D ISB), a two-dimensional (2D) model of an expanding ancestral population that subdivides into two bottlenecked derivative populations, showed the best fit based on low bootstrap variance and high maximum likelihood (LL=−43,587) (see “Materials and methods”; Additional file 1: Figure S12; Additional file 2: Table S27), as illustrated in Fig. 3. The DaDi modeling results imply a >100,000-year-old founder event for cheetahs, perhaps a consequence of their long Pleistocene migration history from North America across the Beringian land bridge to Asia, then south to Africa, punctuated by regular population reduction as well as limiting gene flow through territory protection. Alternatively, Barnett et al. [35] have postulated, based on a study of ancient DNA of *Miracinonyx trumani* (American cheetahs), that today’s African cheetahs originated from Asia, which would indicate that the 10,000-year-old founder effect coincided with an Asia to Africa cheetah dispersal around that time.

More recent late Pleistocene bottlenecks for eastern and southern African populations would further deplete variation in both populations [2, 7, 9]. The AFS modeling indicated a notable excess in derived alleles in the Namibian population compared to the Tanzanian population, implying historic gene flow from Namibian to Tanzanian predecessors estimated at >11,084–12,589 years ago in Africa (Fig. 3; Additional file 1: Figure S12; Additional file 2: Table S28). A parallel analysis using the pairwise sequentially Markovian coalescent (PSMC) algorithm for estimating demographic history lent support to the inference of decreasing cheetah population size in the last 100,000 years (Additional file 1: Figure S11).

**Fig. 4:**
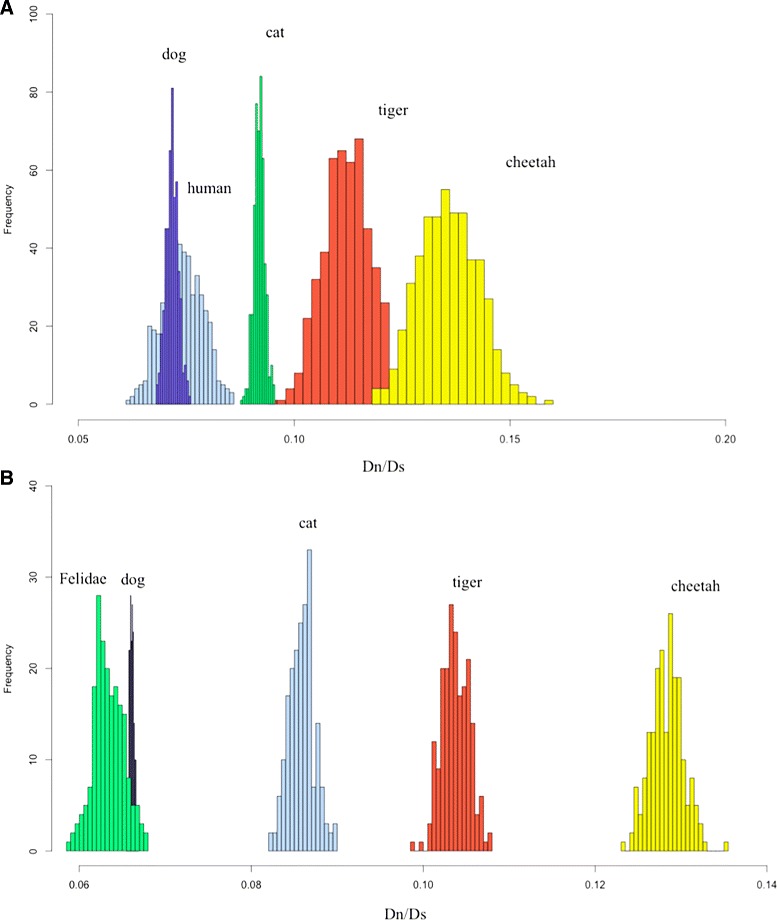
Comparison of Dn/Ds distributions for reproduction-related and all cheetah genes. **a** Distributions of branch-specific values of Dn/Ds for reproductive system genes. Dn/Ds ratios were calculated for five species (dog, human, cat, tiger and cheetah) based on 500 bootstrap replications and the free-ratio model in PAML [37]. **b** Distributions of branch-specific Dn/Ds values for four species (dog, cat, tiger and cheetah) and ancestral reconstructed Felidae branch. Dn/Ds ratios for branches based on 200 bootstrap replications of 10 Mb protein-coding sequences

Modern cheetahs display multiple physiological correlates of inbreeding depression in both captive and free-ranging populations. Compared to other Felidae species, cheetahs show constitutive impairments in reproduction, including low fecundity in captivity, an average of 80 % malformed spermatozoa per ejaculate and an elevated incidence of acrosomal defects, as has been observed in other inbred natural populations [9, 11, 12, 36]. To explore genes that might have mediated the cheetah’s reproductive issues, we first identified 964 human genes with gene ontology (GO) terms related to reproduction, encoding 1730 RNA transcripts. The list was narrowed to 656 genes that had a 1:1 ortholog match among the cheetah, cat, tiger, dog and human based on BLAST and syntenic orthology using Proteinortho/PoFF [37]. We aligned these genes using the parallel tool ParaAT [38] and using PAML to search for genes with an accelerated rate of non-synonymous to synonymous substitution (Dn/Ds) accumulation in the cheetah lineage [37]. Overall, cheetahs displayed a far more accelerated accumulation of non-synonymous mutations relative to other species (Fig. 4). We identified 92 cheetah genes with statistically significant elevated Dn/Ds ratios; for these, we identified the type and frequency of damaging mutations. Eighteen genes had damaging common or invariant constitutive damaging mutations previously implicated in spermatogenesis, azoospermia, oligospermia, gonadal dysfunction and oogenesis (Additional file 2: Tables S29 and S30; Additional file 3: Datasheet S6). Of these, one gene (*AKAP4*) showed an accelerated accumulation of damaging deletions or missense mutations among sampled cheetahs based upon the Polyphen2 database. An alignment of these amino acid sequences showing these potentially deleterious mutations in *AKAP4* of the cheetah compared to orthologs in several other species is presented in Additional file 1: Figure S13. These mutations in *AKAP4* were not observed in the tiger, domestic or wildcat orthologs, nor in the Asiatic Gir lion, a population showing extreme genetic depletion and similar extensive reproductive defects. Sanger sequencing validated four of the five amino acid substitutions in *AKAP4* mutation as homozygous in 10 Namibian cheetahs. The fifth substitution was not validated explicitly. The cheetah’s reproductive gene impairments are strong candidates to explain the compromised reproductive phenotype that afflicts all cheetahs.

A second approach used gene effect annotation in seven sequenced genomes to find harmful mutations segregated in cheetah populations. SNVs showing possible deleterious effects were identified using snpEff [39] and filtered with the names of 656 previously identified 1:1 orthologs from five species related to reproduction gene function and potentially harmful effects (e.g., stop codon gained and affected splice sites). A total of 61 genes were found and 20 of them (Additional file 3: Datasheet S8) showed a primary relationship to the reproductive abnormalities found in cheetahs. These mutations provide a valuable basis for association studies of reproductive impairments in cheetah populations.

**Fig. 5:**
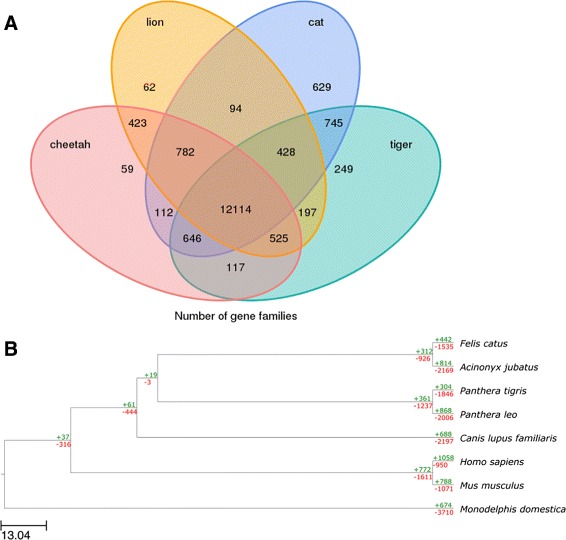
Analysis of orthologous gene families. **a** Unique and shared gene families in the cheetah genome. **b** Dynamic evolution of ortholog gene clusters. The estimated numbers of ortholog groups in the common ancestral species are shown on the internal nodes. The numbers of orthologous groups that expanded or contracted in each lineage after speciation are shown on the corresponding branch, with + referring to expansion and − referring to contraction. The cheetah genome contained 17,863 orthologous gene families. Among these, 10,983 orthologous gene families were shared by all eight genomes and 12,114 by felids while 11 orthologous gene families were exclusively shared among Felidae species (cat, lion, tiger and cheetah) and another 112 were exclusively shared by the cheetah and cat (Additional file 3: Datasheet S2). There were 1335 predicted genes containing 2293 InterPro domains unique to cheetahs (Additional file 3: Datasheet S1). Both figures are based on the comparison of orthologous gene families among eight mammalian species

To extend a detailed annotation of the cheetah genome (Table 1), gene clusters were constructed using eight mammalian genomes (cheetah, tiger, lion, cat, human, dog, mouse and opossum; see “Materials and methods”). The cheetah genome contains 17,863 orthologous gene families. Among these, 10,983 orthologous gene families were shared by all eight genomes and 12,114 by felids, while 112 were shared exclusively by the cheetah and domestic cat (Fig. 5a; Additional file 3: Datasheet S2). There were 1335 predicted genes unique to cheetahs; 812 of them contained 2293 protein domains identified by an InterPro scan [40] (Additional file 3: Datasheet S1). Based on the comparison of orthologous gene families among eight mammalian species, the cheetah genome has 814 expanded and 2169 contracted gene families compared with the feline common ancestor (Fig. 5b).

The expanded genes were largely a variety of GO terms including olfactory and G-coupled protein receptors (also expanded in other Felidae [19, 20, 28]), which, if affirmed, would relate to cheetah physiology. For example, the *LDH-A* and *LDH-B* gene families showed twofold gene number expansions in certain Felidae (cat, cheetah and lion) compared to other mammals, which is potentially explanatory of the Felidae carnivorous life style (Additional file 1: Figure S14).

We searched for signatures of recent natural selection across all cheetah genes by assessing Dn/Ds ratios in alignments with orthologs from the lion, tiger, cat, human and mouse genomes. Specifically, we used the PAML branch-site test to test for positive selection along the cheetah phylogenetic lineage [37] and found 946 genes with significant signals (*p*<0.05 adjusted; Additional file 3: Datasheet S5), ten of which showed enrichment in specific GO terms. Five genes with signatures of selection were related to the regulation of cardiac and striated muscle contraction (*ADORA1*, *RGS2*, *SCN5A*, *ADRA1* and *CACNA1C*); two genes (*TAOK2* and *ADORA1*) were involved with MAPPK activity important in stress response, including heat stress, and four genes (*APOC3*, *DDIT4*, *SUFU* and *PPARA*) were associated with negative regulation of catabolic processes (Additional file 3: Datasheet S5). A copy number variation screen revealed 12.4 Mb included in segmental duplications (SDs) (shared among seven cheetahs) implicating gene regions and plausible gene candidates that might influence cheetah energetics, nutrition and sensory adaptations (Additional file 1: Figures S16 and S17; Additional file 2: Table S32; Additional file 3: Datasheet S7). These selected, expanded or duplicated genes are all possible explanatory candidates for mediating the cheetah’s adaptation to high-speed acceleration and short-term endurance.

## Discussion

African cheetah genomes display a remarkable reduction in endemic genetic variation and footprints of a fascinating natural history. Seven distinct measures show a species losing 90–99 % of variation levels seen in outbred mammals, well below that observed in genome studies of inbred dogs and inbred cats and in genetically depleted Tasmanian devil or Virunga mountain gorilla genomes (Figs. 1 and 2; Additional file 1: Figures S7–S10; Additional file 2: Tables S15–S25). A single exception, the Gir Forest lion population in Gujarat India, is a lion subspecies so inbred that DNA fingerprints of all Gir lions are identical (Fig. 1b; [23, 24]). Cheetahs accept surgically exchanged skin grafts as if they were immunologic clones [9], prompting a study of the cheetah’s MHC. A high-resolution bacterial artificial chromosome (BAC) clone assembly of cat compared to cheetah directly revealed a loss of 2–4 MHC class I genes (*FLA-F*, *-H*, *-I* and *-M*) plus near zero class I amino acid variation across seven cheetah genomes, compared to appreciable domestic cat MHC diversity (Fig. 2; Additional file 1: Figures S9 and S10; Additional file 2: Table S26).

A coalescent demographic analysis (DaDi; [34]) plus a PSMC assessment of genome-wide SNV variation from two African cheetah populations show evidence of two bottlenecks: one ∼100,000 years BP and a second ∼12,000 years BP (Fig. 3). Previous mtDNA and microsatellite imputations also suggested a recent 10–12,000 years BP origin of modern cheetah variation, coincident with the late Pleistocene extinction of predominantly large animals: mammoths, mastodons, dire wolves, short-faced bears, American lions, saber-toothed tigers and four types of flesh-eating birds [1, 2, 25, 41]. Pumas and cheetahs also disappeared from North America at this time [4, 7]. We propose that the two late Pleistocene bottlenecks collapsed diversity in the cheetah’s ancestors and left behind signatures of demographic reduction in their genome sequence. First, ∼100,000 years ago, a migration of cheetahs across Asia and into Africa in a geographic spread possibly originating in North America [2, 4, 7, 8, 35] would have increased incestuous mating as a consequence of behavioral reinforcement of territories during these episodes. The more recent 12,000-year-old founding of African cheetah populations further reduced numbers and led to additional loss of endemic variability observed in modern cheetahs.

Genomic analysis revealed compelling statistical evidence for reproduction gene families accumulating excess functional (amino acid altering) variants in cheetahs, relative to other felids (Fig. 4) and identified ten fixed amino acid variants in the *AKAP4* locus, a gene expressed exclusively in the testis whose homologues play a critical role in sperm development and onset of spermatozoa aberrations in several mammal species [42–44]. Five homozygous function-damaging mutations within *AKAP4* likely would explain the very elevated pleiomorphic sperm (on average 81.6 % damaged spermatozoa) in every cheetah. Certain genes that mediate energy metabolism showed selective acceleration and are candidates for the cheetah’s adaptions to high-speed pursuit. Overall, the cheetah genome offers unparalleled insight into the history, adaptation and survival of a treasured endangered species. The zoo community’s assignment of captive cheetahs as research animals decades ago and the subsequent inclusion of genetic measures in nearly all conservation management deliberations illustrate the continuing benefit from the lessons of the cheetah [5, 10]. In concert with ecological, habitat restoration and other conservation issues, the cheetah’s genetic disposition should be useful in efforts to sustain and increase cheetah population numbers in their present and former range habitats [45].

## Materials and methods

### Sequencing and assembly of the *Acinonyx jubatus* genome

High molecular weight genomic DNA was extracted from blood or tissue samples of seven cheetahs, four from Namibia (one female and three males) and three from Tanzania (one female and two males), using the DNeasy Blood and Tissue kit (Qiagen). The genome of a male Namibian cheetah from the Cheetah Conservation Fund center (Chewbaaka) was sequenced at high coverage on the Illumina HiSeq2000 platform using a shotgun-sequencing approach. Extracted DNA was used to construct short, medium and long mate-pair libraries (170 bp, 500 bp, 800 bp, 2 kbp, 5 kbp, 10 kbp and 20 kbp). Statistics for the obtained reads are given in Additional file 2: Table S1. Six additional samples were sequenced at low coverage (5–6 ×) using 500 bp insert size libraries (Additional file 2: Table S2).

Sequence reads were assembled with SOAPdenovo2 [46], first into contigs and then iteratively into scaffolds with a total genome size of 2.38 Gb and scaffold N50 length of 3.1 Mb (contig N50 length of 28.2 kbp). The genome size was found to be smaller than that based on estimates of the 17-mer length distribution (Additional file 1: Figure S1; Additional file 2: Table S3) [47]. This mismatch may be due to some repetitive sequences or highly complex regions that could not be assembled by the SOAPdenovo2 assembler (Additional file 2: Table S4).

We assessed the sequencing depth distribution and the GC content by mapping all the short insert-size reads back to the high-coverage reference genome and then calculating the GC content and depth for 10-kbp non-overlapping windows along the whole genome (Additional file 1: Figures S2 and S3).

To produce the cheetah chromosome assembly, we mapped cheetah scaffolds using NCBI BLAST [48] onto the domestic cat chromosomes from the *Fca-6.2* assembly, which is based on previously published physical and linkage maps [49]. A summary of the obtained cheetah chromosomes is given in Additional file 2: Table S5.

To find scaffolds that could be associated with the cheetah Y chromosome, we searched human genes located on the Y chromosome in the cheetah scaffolds that were not placed to the cat autosomes or X chromosome using our gene annotation pipeline (see “Annotation of *Acinonyx jubatus* genome” below). Of the 54 protein-coding genes on the human Y chromosome, sequences for 21 genes were predicted in the unplaced cheetah scaffolds (*scaffold1492*, *scaffold1496*, *scaffold1636*, *scaffold803* and *scaffold912*). The *SRY* gene was predicted in the cheetah *scaffold1636*. In total, we found five scaffolds putatively constituting cheetah chromosome Y; their total length was 1,524,629 bp.

### Annotation of *Acinonyx jubatus* genome

#### Repeat annotation

To identify all known Carnivora repeats, we used the RepeatMasker software [50] and the Repbase Update library [51] with the option to search for Carnivora-specific repeats. We searched for repeats in the following genomes: cheetah, lion (*Panthera leo*), tiger (*Panthera tigris* [28]), cat (*Felis catus*; the *Fca-6.2* assembly [19]) and dog (*Canis lupus familiaris*; the *CanFam3.1* assembly; [32]). A summary of the RepeatMasker results are given in Additional file 2: Table S6. In addition, we used the RepeatProteinMask tool, belonging to the RepeatMasker package, which identified transposable elements by aligning a genome sequence to a self-defined transposable-element protein database (Additional file 2: Table S7). To detect tandem repeats in five Carnivora genomes (cat, cheetah, dog, lion and tiger), we used the Tandem Repeats Finder (TRF) software, version 4.07 [52] with the mismatch and maximum period parameters set to 5 and 2000. TRF output was processed as published previously [19].

Observed tandem repeats were divided into three groups:

1. Microsatellites with a monomer length less than 5 bp, including perfect microsatellites with a monomer length of less than 5 bp
2. Complex tandem repeats
3. Large tandem repeats characterized by large successfully assembled tandem repeat arrays that were divided into three subgroups by array length of 1, 3 and 10 kbp (Additional file 2: Tables S8 and S9)

The dog genome contains around 20 % more ascertained tandem repeats and significantly more assembled large tandem repeats in comparison with the four felid genomes.

Complex tandem repeats included large tandem repeats and satellite DNA characterized by GC content of arrays from 20 to 80 %, array length greater than 100 bp, copy number variations greater than 4 bp in length, array entropy greater than 1.76, monomer length greater than 4 bp, and imperfect tandem repeat array organization. Complex tandem repeats were classified into families by sequence similarity computed using NCBI BLAST according to the workflow from [19]. Each family was named according to nomenclature based on the most frequent monomer length. The family *Ajub483A* is the most similar to the FA-SAT repeat of the domestic cat and it has predicted locations in the pericentromeric and pretelomeric regions [53, 54]. Families *Ajub33A* and *Ajub113A* have predicted locations in the pericentromeric regions. Family *Ajub84A* is based on the tandemly repeated zinc-finger motif (Additional file 2: Table S9).

#### Gene annotation

In total, 20,343 protein-coding genes and 110,431 (10.1 Mb) non-coding RNA elements were identified in the cheetah genome (Additional file 2: Tables S10 and S11).

**Coding genes** To predict the protein-coding genes in the cheetah, we combined both homology-based and de novo gene prediction tools. We first downloaded the gene sets from Ensembl (http://www.ensembl.org) [55] for the cat, dog and human and chose the unique locus for each gene by extracting the longest open reading frame for the multi-open-reading-frame genes. We then used the NCBI BLAST tool [48] with an E-value cutoff of 10^−5^ for mapping all orthologous genes onto the reference cheetah genome in an effort to speed up alignment. We also used Genewise [56] to carry out local alignment and predict a gene structure for each possible linked orthology hit. Genes that were complete both in terms of structure and in length based on the orthology searches were then used as input to train the hidden Markov gene model to predict also gene structure using the Augustus software package [57]. If a conflict was found between the orthology-based and de novo prediction methods, we used the gene result based on the orthology-based methods alone.

**Non-coding RNAIdentification of tRNA genes** The tRNA genes were predicted by tRNAscan-SE [58] with eukaryote parameters. If more than 80 % length of a tRNA gene was covered by the transposable small interspersed elements (SINE), then it was defined as SINE-masked. The tRNA identity to human was calculated with a MUSCLE [59] global alignment.

**Identification of rRNA genes** The rRNA fragments were identified by aligning the rRNA template sequences from the human genome using BlastN [48] at *E*-value 10^−5^, with a cutoff of identity ≥85 % and match length ≥50 bp.

**Identification of other ncRNA genes** The miRNA and snRNA genes were predicted using the INFERNAL [60] software against the Rfam database (release 9.1, 1372 families) [61] with Rfam’s family-specific “gathering” cutoff. To accelerate the speed, we performed a rough filtering prior to INFERNAL by aligning the obtained miRBase predictions against the Rfam sequence database using Blastn under an *E*-value of 1. The miRNA predictions were first aligned against the mature sequences of human and dog from miRBase [62] (release 13), allowing one base mismatch, and then aligned against the precursor sequences, requiring more than 85 % overall identity. The snoRNA predictions were aligned to human H/ACA and C/D box snoRNAs and Cajal body-specific scaRNAs from snoRNABase [63] (version 3), and required a cutoff of 85 % overall identity. The spliceosomal RNA predictions were aligned to the Rfam sequence database, and required a cutoff of 90 % overall identity.

#### SNV annotation

To increase the sample size (power) for genome variation and population analyses, we combined the reads from the six re-sequenced cheetah genomes with the reads from the reference cheetah genomes using only 500-bp insert size libraries for all individuals. Therefore, our population genomic analyses are based on seven individual cheetahs, four from Namibia and three from Tanzania.

**Raw reads filter and mapping** The reads were subject to quality control measures using an in-house Perl script. The procedure removed all full or partial low-quality reads that met one or more of the following criteria:

1. An N-content of more than 10 %
2. More than 40 % of the read length was below Q7
3. Reads overlapping by more than 10 bp with an adapter sequence, with a maximum of 2 bp mismatches
4. Paired-end reads, which overlapped by more than 10 bp between the two ends
5. Duplicate reads

We observed that both ends of a read, with total length equal to 90 bp, always had low quality scores, especially the 3^′^ end. We, therefore, trimmed a maximum 10 bp off the 5^′^ end of a read if the consecutive quality score was less than Q20. Likewise, we trimmed a maximum of 40 bp off the 3^′^ end of a read if the consecutive quality score was less than Q20. In this way, we retained enough bases with high quality for the subsequent read mapping.

We used the Burrows–Wheeler aligner [64] to map the raw reads onto the assembled reference genome, with the option -e 10, which allows a maximum ten-gap extension in a hit. The remaining arguments were run with the default settings. We further filtered the Burrows–Wheeler alignments for the subsequent single-nucleotide polymorphism, calling according to the following criteria:

1. Alignments with a mapping quality score less than 20 (<MQ20)
2. Non-unique alignments, i.e., any alignments that mapped to multiple positions in the genome sequence
3. Duplicated alignments, i.e., two or more reads that aligned to exactly the same position in the genome sequence

**SNV calling using the site frequency spectrum method** SNV calling based on low-depth sequencing (< 10×) is a challenge for most of the current strategies. The method described in [65] is a robust and high-precision method for SNV calling at low depth based on the methodology of the site frequency spectrum (SFS). It uses a maximum likelihood algorithm to estimate the maximum probability for each site. We used the SOAPsnp method to produce the GLFv2 format for each site and then used ANGSD [65] and beagle [66] to extract the genotype.

Initially, we obtained the SNV list for high-coverage sites across the whole genome in which the minimum and maximum read depths for each sample were set to 5 × and 30 ×, respectively (Additional file 1: Figure S4). Finally, 3.44 million SNVs were ascertained (Additional file 2: Table S15). In addition, variant positions located in repeat regions were filtered out, which produced a final set of 1,820,419 SNVs, which is 53 % of the original SNV number (3,438,824). We ascertained the distribution of SNVs across the genome for all individuals (Additional file 2: Table S20). All SNV variants were annotated for each individual using snpEff [39] and a database was constructed from the annotated cheetah genes (Additional file 1: Tables S16–S18). For all observed SNVs, 73.7 % were located outside the protein-coding genes; only 1.3 % were inside exons and a major fraction of them, 24.92 %, were found inside introns (Additional file 2: Table S19).

#### Nuclear mitochondrial segments

We retrieved copies of nuclear mitochondrial segments using the whole *Felis catus* cytoplasmic mtDNA genome (RefSeq:NC_001700) as the query input sequence in an NCBI BLAST search. This search found 143 sequences with significant identity to the cat mtDNA genome, 50 of which contained complete mitochondrial genes and 93 partially covered genes (Additional file 2: Table S12).

#### Mitochondrial genome assembly and nucleotide diversity analysis

Complete mitochondrial genomes of all seven cheetahs were assembled using the 500-bp insert libraries from the reference and re-sequenced individuals. There is very little variation among the cheetah sequences (∼0.1–0.2 % divergence across the entire mitogenome). There does appear to be variation that separates the eastern versus southern African cheetah populations.

Additionally, nucleotide diversity was examined in a number of other mammalian species and compared with the cheetah. Cheetahs have the lowest numbers in diversity among other species, and numbers are correlated with population sizes for Tanzania and Namibia (Additional file 2: Table S25).

#### Genome rearrangements

**Whole-genome alignment** We used the Progressive Cactus software [21, 67] to align the scaffold assemblies of the tiger, lion and cheetah, and the chromosome assemblies of the domestic cat (*Fca-6.2*) and domestic dog (*CanFam3.1*). The dog genome was included as an outgroup. The percentage alignment of the cheetah genome to the other genomes was 93.6 % for the tiger, 91.6 % for the lion, 91.1 % for the domestic cat and 74.1 % for the domestic dog.

**Calculation of synteny blocks** The multiple alignment was further processed with the GRIMM synteny algorithm [22, 68]. The aligned segments of the genomes are used as anchors that are further chained into syntenic blocks. The size of the blocks is a flexible quantity and can be controlled by the input parameters that correspond to the minimal size of a block and maximal proximity between the aligned anchors that will be joined into one cluster. We set both parameters equal to 300 kbp because other variants produced many short syntenic blocks and these parameter values were shown to be optimal in previous analyses of the human and mouse [69]. For each synteny block, we calculated the density of anchors. Density is defined as the sum of lengths of aligned anchors divided by the length of the whole block [69]. After filtering out those syntenic blocks that correspond only to single scaffolds in the cheetah genome, 93 syntenic blocks remained, which were used for further analyses. The ten longest syntenic blocks showing rearrangements are shown in Additional file 1: Figure S6.

**Calculation of genome rearrangement scenarios** We applied the GRIMM algorithm [22, 68] to the synteny blocks to calculate the rearrangement scenarios that occurred between the cheetah and each of the other four species. Since we used scaffold assemblies for the cheetah, tiger and lion, we needed to distinguish rearrangement events that occurred in the separate scaffolds from those that occurred within the scaffolds of each species. The synteny blocks between the cat and cheetah genomes cover the largest fraction of the cheetah genome (98.6 %) (Additional file 2: Table S13), likely because the domestic cat genome assembly is more complete compared to the assemblies of the lion and tiger. The results also agree with the relatively short evolutionary distance between the cat and the cheetah, 6.7 MY [70]. For comparison, the synteny blocks in the human–mouse alignment cover 82 % of the human genome [71], where the divergence time for human and mouse is 96 MY.

We also analyzed the distribution of the syntenic block lengths for the blocks for which the length was greater than 10 kbp (Additional file 1: Figure S5). The peaks in the graph correspond to the number of synteny blocks with the corresponding length. The plots demonstrate that there are more syntenic blocks of shorter lengths than those of the longest one. We found that the lion genome is the most fragmented, which explains why most cheetah–lion synteny blocks have a length <1.5 Mb. The graphs for the cat and dog are similar, with syntenic blocks that are longer compared to the lion and tiger due to the higher assembly quality of the former two species. With the GRIMM software, we also calculated the rearrangement scenarios based on the multiple alignments (Additional file 2: Table S14). The results of this approach can be verified by PCR amplification.

### Gene evolution in *Acinonyx jubatus*

#### Gene family clusters

For the gene family analyses, we used eight mammalian species, including four felids: human, mouse, dog, opossum, domestic cat, cheetah, lion and tiger. DNA and protein data for five mammals (human, mouse, dog, domestic cat and opossum) were downloaded from the Ensembl database (release 56). For genes with alternative splicing variants, the longest transcripts were selected to represent the genes. We used the methodology implemented in Treefam [72] to define a gene family as a group of genes descended from a single gene in the last common ancestor of the considered species. This procedure was conducted in two steps:

1. Blastp was applied to align all protein sequences against a database containing a protein data set of all species, with the *E*-value set to 10^−7^ and with -outfmt 6. In addition, fragmented alignments were joined for each gene pair using Solar (perl solar.pl -a prot2prot -f m8 -z). We assigned a connection (edge) between two nodes (genes) if more than 1/3 of the region aligned to both genes. An Hscore that ranged from 0 to 100 was used to weight the similarity (edge). For two genes, *G*_1_ and *G*_2_, the Hscore was defined as *sc*(*G*_1_,*G*_2_)/ max(*sc*(*G*_1_,*G*_1_),*sc*(*G*_2_,*G*_2_)), where *sc* is the BLAST bit score.
2. Gene families were clustered using Hcluster_sg with options set to -w 10 -s 0.34 -m 500 -b 0.1. We used the average distance for the hierarchical clustering algorithm, requiring the minimum edge weight (Hscore) to be larger than 5, and the minimum edge density (total number of edges/theoretical number of edges) to be larger than 1/3. Clustering for a gene family would stop if it already had one or more of the outgroup genes.

To determine the expansion and contraction of the orthologous protein families among nine mammalian species, we used CAFE 3.0 [73] with its lambda option (the gene gain and loss rate) set to 0.0024. GO enrichment analyses were used to test for overrepresented functional categories among expanded genes and genome-background genes (Additional file 3: Datasheets S3 and S4). All results with a *p* value higher than 10^−4^ were filtered out. Also the false discovery rate was calculated to take into account multiple testing.

#### Positively selected genes

To detect genes that evolved under positive selection, we used PAML, a maximum-likelihood method for analysis of molecular evolution [37, 74]. Specifically, we used PAML’s branch-site test [75] to test for positive selection along the cheetah lineage. We compared model A1, in which sites may evolve neutrally and under purifying selection with model A, which allows sites to be also under positive selection. *p* values were computed using the *χ*^2^ statistic adjusted using the false discovery rate [76] to allow for multiple testing. Alignment quality is of major importance for studies of positive selection, as alignment errors can lead to unacceptably high false positives using the branch-site model [77]. We used PRANK [78], which differs from other alignment tools in that it utilizes evolutionary information in determining where to place a gap. Studies of the branch-site test and other PAML models support PRANK to be the alignment tool of choice [77, 79]. We filtered the PRANK alignments by Gblocks [80, 81] and excluded genes with sequence properties that often lead to false positives, such as genes with a high proportion of low complexity or disordered regions, ubiquitous domains, repeats, transmembrane and coiled-coil regions, overlapping domains, uncharacterized proteins, collagens, Zn-finger proteins, olfactory receptors and other large families or clustered arrangements. We identified 947 genes (Additional file 3: Datasheet S5) under positive selection (*p*<0.05 adjusted for multiple testing). Of the 947 genes that showed signals of positive selection in the cheetah lineage, seven genes were selected during GO analysis (the maximum *p* value was 10^−3^), which we found were related to regulation of muscle contraction (*ADORA1*, *RGS2*, *SCN5A*, *ADRA1B*, *CACNA1C*, *TAOK2* and *SCAI*), and which exhibit an important role in cheetah locomotion and cardiac muscle contraction, and two genes (*TAOK2* and *ADORA1*) associated with MAPKK activity, which is important in the stress response, including heat stress (Additional file 3: Datasheet S5).

### Analysis of reproduction-related gene families in *Acinonyx jubatus*

To analyze reproduction-related genes in the cheetah genome, we obtained human genes belonging to the gene ontology term GO:0000003 (Reproduction) from the Ensembl Genes database [55]. A total of 1730 transcripts of 964 protein-coding genes were obtained. This set was used to find 1:1 orthologous genes in the cheetah, cat, tiger, dog and human. To find orthologous relationships between genes, the method Proteinortho/PoFF [82], which utilizes both BLAST alignment and synteny approaches, was used. Of the 1730 transcripts, the search resulted in 656 1:1 orthologs for the five species.

Orthologs were aligned using the parallel tool ParaAT [38] with the MAFFT aligner [83] with the options set to the most accurate, taking into account absent exons in some genes. To delete putatively misaligned regions, Gblocks [81] was applied to the multiple sequence alignments with stringent filtering criteria; the following Gblocks parameters were used: -b1=5 -b2=4 -b3=6 -b4=10 -b5=h.

We used PAML to find genes with an accelerated accumulation of non-synonymous to synonymous rates (Dn/Ds) in the cheetah lineage relative to the mean in the four species. An accelerated accumulation of non-synonymous substitutions may indicate an increased number of moderate and deleterious mutations that are harmful for the reproductive physiology in the cheetah lineage. To estimate the rate of non-synonymous mutation accumulation, the free-ratio model implemented in PAML was used [74]. The model assumes a different lineage-specific rate of the Dn/Ds ratio for each branch of the tree. All genes were concatenated into one “supergene” and Dn/Ds was estimated for each species. Surprisingly, the cheetah had the highest values for Dn/Ds rate among the five studied species. To test this effect further, a new data set was generated using 500 bootstrap replications (Fig. 4a).

To test the hypothesis that there are elevated Dn/Ds values in the cheetah lineage, the total set of 6348 genome-wide orthologs was constructed for all genes from the following species: cat, tiger, cheetah and dog. After filtering unreliably aligned regions using Gblocks and concatenation, a 10-Mb long alignment of coding sequences was obtained. Based on the alignment, 200 bootstrap replications were performed and the resulting data set was used for the free-ratio analysis in PAML. For the whole genome data set, the same results as given above were obtained (Fig. 4b); the cheetah had accelerated Dn/Ds ratio values relative to the other species.

To find genes with elevated Dn/Ds ratios in the cheetah lineage associated with reproduction (e.g., oogenesis and spermatogenesis), the branch-site test was performed for each of the 637 genes (the properly aligned set from the 656 1:1 orthologs we originally found) using the following two models:

1. M0—Same Dn/Ds for all branches of the tree
2. M2—Different Dn/Ds for background (cat, human, dog and tiger) and foreground (cheetah) branches

All genes with Dn/Ds ratio values in the cheetah branch greater than those in the other branches based on the M2 model were retrieved from the whole data set and the likelihood ratio test between the M0 and M2 models was performed (to test the hypothesis that the Dn/Ds ratio is significantly greater in the cheetah lineage compared to the other species). In total, 92 genes with *p*<0.05 were obtained (Additional file 3: Datasheet S6). These genes were manually screened using public databases (GeneCards and Ensembl) to find genes directly related to spermatogenesis, azoospermia, oligospermia, oogenesis and gonadal dysfunction. A final list of 18 genes was used (Additional file 2: Table S29) to search the genetic-disease databases, including OMIM, KEGG and MalaCards, as well as to screen for all non-synonymous mutations and deletions. The pathogenicity of mutations was assessed using PolyPhen2 [84] with human proteins as the model for the cheetah. Among the 18 genes, we discovered one gene that showed an excess of possibly damaging missense mutations and was related to important spermatogenesis functions: *AKAP4*. We used Sanger sequencing to validate *AKAP4* mutations in 10 Namibian cheetahs. Four from the five non-synonymous substitutions were confirmed in 9 samples and appeared to be homozygous. The fifth mutation was not detectable as it was located in one of the primer sequences.

### Analyses of genetic diversity in the *Acinonyx jubatus* genome

SNV diversity was analyzed for the seven cheetahs and compared with SNV diversity in four other species: domestic cat, Bengal tiger, Siberian tiger and African lion. We constructed 50-kbp windows from the 3802 cheetah scaffolds, which were used to estimate SNV density at each window. Of these, 2386 scaffolds had lengths less than the specified window size and thus, were excluded from further analysis; most of these fragments were contigs with length less than 500 bp. The remaining 46,787 windows used had a total length of 2.34 Gb. Altogether, the windows constituted 99.12 % of the total length of the genome. The number of genes with SNVs located in the coding sequences (exons) was also examined for SNV density and compared among species (Additional file 2: Tables S20–S24).

Runs of homozygosity were estimated following the method described in [85] and using PLINK with the following parameters: –homozyg-window-snp 20–homozyg-density 50–homozyg-kb 10. Genome-wide heterozygosity was estimated by splitting the whole genome into non-overlapping windows of 100 kbp and counting the number of SNVs in them. Next, a window was considered heterozygous if the number of SNVs in it was greater than 40, otherwise it was considered homozygous. In Additional file 1: Figures S8a–S8d, the distribution of homozygous and heterozygous windows is shown for Boris (an outbred domestic cat), Cinnamon (an inbred domestic cat), Chewbaaka (a cheetah) and the mountain gorilla individual [86], respectively.

### Demographic history analyses of the *Acinonyx jubatus* population

#### Pairwise sequentially Markovian coalescent analysis

We used the PSMC method [87] to infer the effective population size trajectory through time of the high-coverage cheetah genome (Chewbaaka). We used the Burrows–Wheeler aligner [64] and samtools [88] for mapping and genotyping. The generation time was set to 3 years and the mutation rate to 0.3×10^−8^, which was based on the whole-genome alignment between the cheetah and domestic cat generated using LASTZ [89] and calculating the number of differences between the two species and dividing by their divergence time (7 MY).

The PSMC results showed a gradual reduction in effective population size through time without any evidence for a sharp bottleneck (Additional file 1: Figure S11). These results may be due to the PSMC analysis having lower sensitivity for events during the more recent past and/or that any bottleneck event was short and severe, leaving little or no trace in the genome.

#### Diffusion approximation for demographic inference (DaDi) analysis

For the two cheetah populations analyzed (southern in Namibia and eastern in Tanzania), the AFS corresponds to a multidimensional matrix *X*, where each *x*_*ij*_ entry gives the number of SNVs with an observed derived allele count of *i* in population 1 (Namibia) and *j* in population 2 (Tanzania). The likelihood is computed, given the expected AFS under a given evolutionary model. Each entry in the expected AFS reflects the probability of a given SNV falling into that cell. Assuming that all SNVs are n (that is, assuming free recombination between SNVs), these probabilities can be derived from the distribution of allele frequencies of each population, which in turn can be found with diffusion approximations of evolutionary processes, such as the size and timing of demographic changes.

To infer the demographic history of the two cheetah populations, we used the DaDi tool [34]. Briefly, DaDi can generate a site AFS under one or more demographic scenarios. The aim is then to maximize the similarity between the expected allele frequency and the observed SFS over the parameter value space. Fitting can be evaluated by computing a composite-likelihood among different demographic scenarios.

Using the AFS of the two cheetah populations, which included ancestral state information, we tested models under five different demographic scenarios to determine which model had the highest likelihood fit with the observed cheetah AFS. To investigate the timing and relationship between the splitting of the ancestral population and bottleneck events, we tested four 2D models:

1. The ancestral population splits into two subpopulations followed by limited migration from one subpopulation to another (2D IM model).
2. The ancestral population first undergoes a bottleneck, followed by splitting into two subpopulations (2D BIM model).
3. The ancestral population first splits into two subpopulations followed by a bottleneck event and then there is expansion/recovery of each subpopulation (2D SBR model).
4. The ancestral population first grows in size for ∼100,000 years prior to splitting into two subpopulations, followed by an independent bottleneck in each subpopulation (2D ISB model).

These four models were independently simulated and their likelihood calculated to compare the fit of each model to the cheetah AFS data. To determine whether the model with the highest likelihood is appropriate for our data, we used two metrics to compare the joint AFS for the Namibia and Tanzania populations with that expected under our simulated scenario:

1. A log-likelihood ratio test using the chi-square test for significance (Additional file 2: Table S27)
2. The variance of the result estimated by 100 bootstrap iterations from randomly selected real data (Additional file 1: Figure S12)

### Segmental duplication analysis in the *Acinonyx jubatus* genome

To estimate regions of recent SDs from the genomes of six *Acinonyx jubatus* individuals, we applied an approach based on genome-wide differences of depth of coverage [71].

**Reference assembly preparation** Regions detected by RepeatMasker [50] and TRF [52] were masked to remove most of the repetitive regions present in the assembly. We further sought to identify and mask potential hidden repeats by using a kmer-based approach. Scaffolds and contigs were partitioned into kmers of 36 bp (with adjacent kmers overlapping by 5 bp) and these kmers were mapped to the assembly using mrsFast [90] to account for multi-mappings. Overrepresented kmers, defined as those with three or more mappings into the assembly, were additionally masked (Additional file 1: Figure S15; Additional file 2: Table S31). For subsequent analysis, we created a shortened version of the assembly that did not include scaffolds or contigs below 10 kbp since we require SDs to expand at least this length because of the lower coverage of the genomes.

**Read mapping and detection of copy number variation** After checking the overall quality of the raw sequencing data, we split the reads into two consecutive kmers of 36 bp corresponding to positions 10–46 and 46–81. We chose the offsets in such way to trim regions of potentially lower-quality reads. These kmers where then mapped with mrFast [90] to the cheetah scaffolds masked by RepeatMasker and TRF (Additional file 2: Table S31) on which an additional 36 bp flanking each masked segment (referred to as padding regions below) were masked. The reason for the introduction of additional padding regions is that copy number variations are detected using mrCaNaVar [71] via the read depth in non-overlapping windows of 1 kbp of the unmasked sequence; i.e., the genomic coordinates of these windows may exceed 1 kbp, as they include masked regions. Reads originating from a region that overlaps a masked segment will not be mappable onto the genome and might, therefore, lead to a drop-off in estimates of the coverage of those positions. To avoid this bias, paddings the size of a split read were introduced.

A genome-wide read depth distribution was calculated by iteratively excluding windows with the most extreme read depth (RD) values and retaining the remaining windows as control regions. The copy number (CN) of any given window was then calculated as CN=2×RD/ mean(RD in control regions). The distribution of copy number values in control regions centered then to the value of 2 (Additional file 1: Figure S16).

**Calling duplication blocks** We define an SD as a region constituted of at least five consecutive windows of a non-overlapping non-masked sequence with CN > mean CN(control regions) + 3 standard deviations, allowing for one of those windows to have a CN value above mean + 2 standard deviations. The cutoffs were defined per sample. Additionally, these windows were to span at least 10 kbp in genomic coordinates. Furthermore, regions with an absolute copy number above 100 in any sample were excluded. For the downstream analysis, we additionally excluded gaps from the called intervals. Furthermore, we did not consider scaffolds that putatively derive from sex chromosomes.

We found a total of 7.8 Mb of the cheetahs’ autosomal genome to be composed of SDs across the six analyzed genomes. Duplicated regions for each individual range from 4.4 to 5.4 Mb and are summarized in Additional file 2: Table S32. About half of these regions (2.4 Mb) are shared by all individuals, despite the relatively low coverage, which may decrease our power to detect SDs. Still, these numbers are still reasonably similar to the ones reported for the domestic cat (9.1 Mb in duplications and 4.3 Mb in shared duplications) [20].

We intersected shared duplicated regions with gene annotations, requiring at least 60 % of the feature to overlap the duplications to be considered. In this way, we identified coding sequences of 173 predicted genes fixed as potential duplications in all individuals. A full list of the identifiers can be found in Additional file 3: Datasheet S7. An example of a fixed duplication intersecting coding regions can be found in Additional file 1: Figure S17. We performed a simple online GO-term enrichment analysis (http://amigo.geneontology.org/rte) with the human parent identifiers or the human orthologs of parent identifiers of genes in fixed duplication and found ontologies associated with smell, sensory perception, stimulus detection and catabolic processes to be enriched.

### Software used in study

Besides the programs mentioned above and in the main text, we also used the following computational tools in our study:

- parallels [91] for parallelizing computations
- Circos [92] for producing circular plots of genome regions and their annotations
- bedtools [93] for processing genome annotation data
- vcftools [94] for manipulating genome variation data

### Availability of supporting data

The data can be accessed through BioProject accession numbers PRJNA297632 for the whole-genome sequence and PRJNA297824 for the re-sequence data. The SRA for whole-genome sequencing can be accessed via reference numbers: SRR2737512, SRR2737513, SRR2737514, SRR2737515, SRR2737516, SRR2737517, SRR2737518, SRR2737519, SRR2737520, SRR2737521, SRR2737522, SRR2737523, SRR2737524, SRR2737525, SRR2737526, SRR2737527, SRR2737528, SRR2737529, SRR2737530, SRR2737531, SRR2737532, SRR2737533, SRR2737534, SRR2737535, SRR2737536, SRR2737537, SRR2737538, SRR2737539, SRR2737540, SRR2737541, SRR2737542, SRR2737543, SRR2737544, SRR2737545. This whole-genome shotgun project has been deposited at DDBJ/EMBL/GenBank under the accession LLWD00000000. The version described in this paper is LLWD01000000.

## Additional files

Additional file 1
**Supplemental figures.** **Figure S1.** Cheetah genome size estimation by 17-mers. **Figure S2.** Depth distribution of cheetah reads. **Figure S3.** GC content and average sequencing depth values. **Figure S4.** Depth distribution of re-sequencing reads. **Figure S5.** Distribution of syntenic blocks in genome windows. **Figure S6.** Ten largest cat-cheetah rearrangements. **Figure S7.** Size of homozygosity stretches in Felidae genomes. **Figure S8.** Ideograms of homozygosity regions. **Figure S9.** Comparison of cheetah and human MHC regions. **Figure S10.** Comparison of cheetah and dog MHC regions. **Figure S11.** Inferred historical population sizes by PSMC analysis. **Figure S12.** Bootstrap values for DaDi demographic models. **Figure S13.** Alignments of the *AKAP4* gene. **Figure S14.** Evolutionary history of LDH gene families. **Figure S15.** Cumulative distribution of 36-mers. **Figure S16.** Copy-number distribution in control regions. **Figure S17.** Example of fixed duplications on scaffold606. (PDF 2027 kb)

Additional file 2
**Supplemental tables.** **Table S1.** Sequenced cheetah reads for de novo genome assembly. **Table S2.** Re-sequenced cheetah reads for population analyses. **Table S3.** Estimated cheetah genome size. **Table S4.** Cheetah genome assembly information. **Table S5.** Reference-assisted assembly of cheetah chromosomes. **Table S6.** RepeatMasker results for transposable elements in carnivore genomes. **Table S7.** Total length of repeat regions in cheetah. **Table S8.** Tandem repeats in five carnivore genomes. **Table S9.** Complex tandem repeat families. **Table S10.** Protein-coding gene annotation. **Table S11.** Non-coding RNA annotation. **Table S12.** Nuclear mitochondrial genes. **Table S13.** Lengths of cheetah synteny blocks. **Table S14.** Cheetah rearrangements. **Table S15.** Called SNV statistics. **Table S16.** SNV effects by impact. **Table S17.** SNV effects by functional class. **Table S18.** SNV effects by genomic region. **Table S19.** SNV locations relative to genes. **Table S20.** SNV distribution in cheetah genome. **Table S21.** SNV distribution in tiger genomes. **Table S22.** SNV locations and effects in coding genes of Felidae genomes. **Table S23.** SNV counts in genes in domestic cat and tigers. **Table S24.** SNV counts in genes in cheetahs. **Table S25.** Nucleotide diversity in mitochondrial genomes of mammals. **Table S26.** Nucleotide diversity in MHC class I and II genes. **Table S27.** Demographic models and their log-likelihood values. **Table S28.** Population data by DaDi. **Table S29.** Reproductive system genes with identified function. **Table S30.** Filtration of cheetah reproduction system genes. **Table S31.** Nucleotide diversity of masked assemblies. **Table S32.** Statistics on autosomal segmental duplications. (PDF 127 kb)

Additional file 3
**Supplemental datasheets.** **Datasheet S1.** List of cheetah-specific de novo predicted genes with functional domains annotated by InterPro scan. **Datasheet S2.** List of gene families in eight mammal species identified by protein homology. **Datasheet S3.** Results of gene family expansion and contraction analysis. **Datasheet S4.** CAFE results from gene family contraction and expansion analysis. **Datasheet S5.** Results of gene selection analysis. **Datasheet S6.** Reproductive system genes with damaging mutations. **Datasheet S7.** Segmental duplication genes. **Datasheet S8.** List of reproductive genes with segregated high effect mutations and corresponding genotypes of cheetah. (XLSX 711 kb)
