# Supplementary material for: Genomic legacy of the African cheetah, Acinonyx jubatus
Source: Genome Biol. 2015 Dec 10;16:277. doi: 10.1186/s13059-015-0837-4 (PMC4676127; doi:10.1186/s13059-015-0837-4)
Supplement: Additional file 1 — Supplemental figures. Figure S1. Cheetah genome size estimation by 17-mers. Figure S2. Depth distribution of cheetah reads. Figure S3. GC content and average sequencing depth values. Figure S4. Depth distribution of re-sequencing reads. Figure S5. Distribution of syntenic blocks in genome windows. Figure S6. Ten largest cat-cheetah rearrangements. Figure S7. Size of homozygosity stretches in Felidae genomes. Figure S8. Ideograms of homozygosity regions. Figure S9. Comparison of cheetah and human MHC regions. Figure S10. Comparison of cheetah and dog MHC regions. Figure S11. Inferred historical population sizes by PSMC analysis. Figure S12. Bootstrap values for DaDi demographic models. Figure S13. Alignments of the AKAP4 gene. Figure S14. Evolutionary history of LDH gene families. Figure S15. Cumulative distribution of 36-mers. Figure S16. Copy-number distribution in control regions. Figure S17. Example of fixed duplications on scaffold606. (PDF 2027 kb) [file 13059_2015_837_MOESM1_ESM.pdf]

Supplemental Figures
for
Genomic Legacy of the African Cheetah, *Acinonyx jubatus*

List of Figures

Figure S1: Cheetah genome size estimation by 17-mers 3
Figure S2: Depth distribution of cheetah reads 3
Figure S3: GC content and average sequencing depth values . . . 4
Figure S4: Depth distribution of re-sequencing reads 4
Figure S5: Distribution of syntenic blocks in genome windows . . 5
Figure S6: Ten largest cat-cheetah rearrangements 6
Figure S7: Size of homozygosity stretches in Felidae genomes . . 7
Figure S8: Ideograms of homozygosity regions 8
Figure S9: Comparison of cheetah and human MHC regions . . . 9
Figure S10: Comparison of cheetah and dog MHC regions 10
Figure S11: Inferred historical population sizes by PSMC analysis 11
Figure S12: Bootstrap values for DaDi demographic models 12
Figure S13: Alignments of the *AKAP4* gene 13
Figure S14: Evolutionary history of LDH gene families 14
Figure S15: Cumulative distribution of 36-mers 15
Figure S16: Copy-number distribution in control regions 16
Figure S17: Example of fixed duplications on scaffold606 17

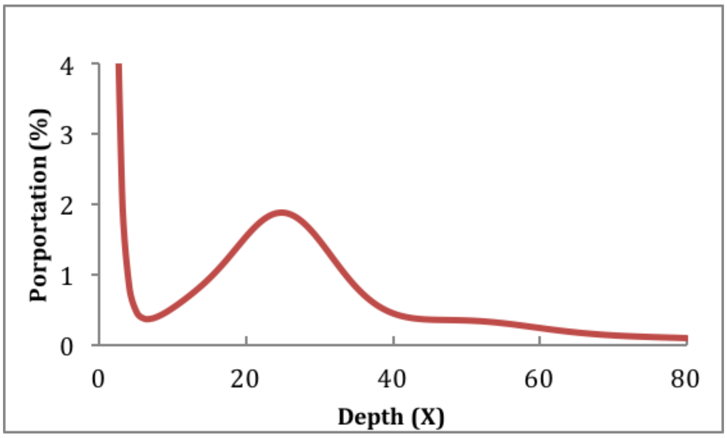

Figure S1: **17-mer coverage distribution.** The distribution was used to estimate the cheetah genome size using the method introduced in [1]. The x-axis is 17-mer coverage depth (that is, occurrence of a 17-mer in the cheetah genome); the y-axis is the percentage of the total number of 17-mers.

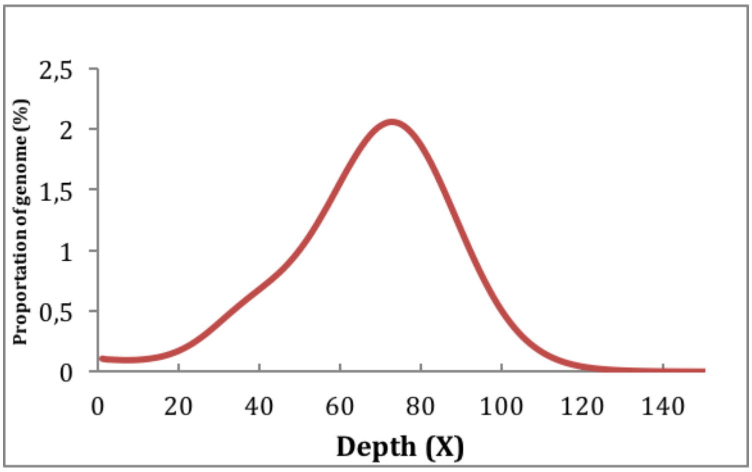

Figure S2: **Distribution of read depth for the de novo assembled cheetah genome.** The x-axis is the number of reads aligned to a genome position (nucleotide); the y-axis is the percentage of such genome positions. The mean read depth value is 73×.

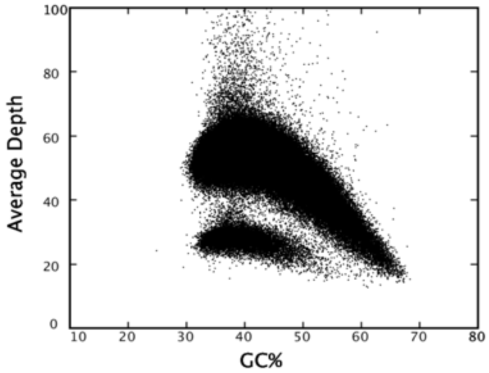

Figure S3: **GC content and average sequencing depth values for cheetah reads.** The assembled cheetah genome was split into non-overlapping windows of 10 kbp. The x- and y-axes represent GC content and average depth values, respectively, for each genome window. The dominant GC content value lies within 35–40%. Also we can see the subgroup (the lower cloud with depth 20–40 and GC 30–50%) of a half depth of the major, which may represent the sex-related chromosome.

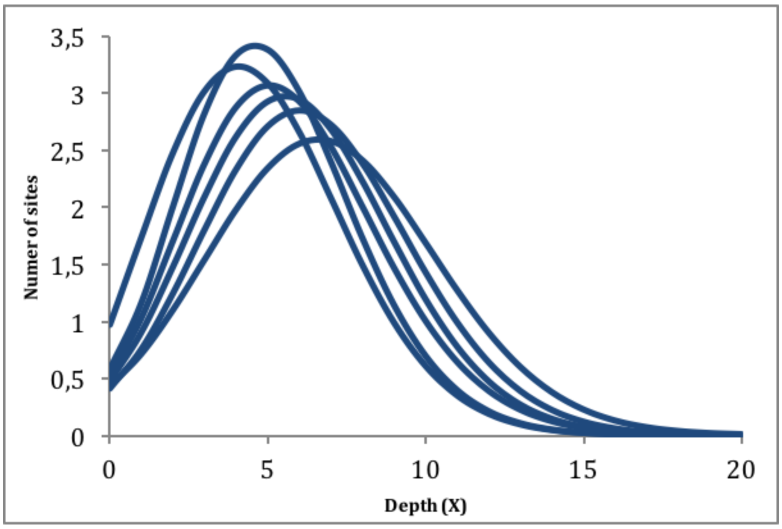

Figure S4: **The depth distribution of re-sequencing reads for all six cheetahs.** The figure is based on short read alignments to de novo assembled cheetah scaffolds. Observed variants were not filtered by coverage or quality. The total number of raw SNV sites is $\sim 8 \times 10^6$.

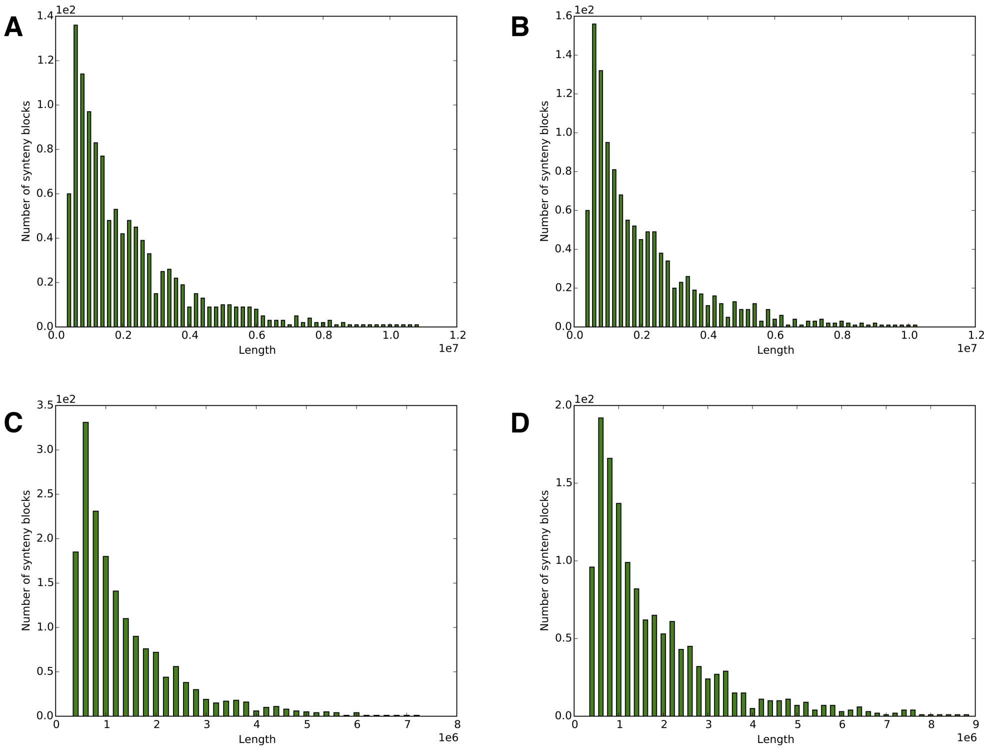

Figure S5: **Distribution of the syntenic block numbers in non-overlapping 10 kbp windows in the cheetah genome.** Synteny blocks relative to four carnivora species were considered: cat, cheetah, dog and lion. The x-axis indicates the number of genome windows containing the number of synteny blocks specified by values on the y-axis. (A) Cheetah-cat synteny blocks (B) Cheetah-dog synteny blocks (C) Cheetah-lion synteny blocks (D) Cheetah-tiger synteny blocks.

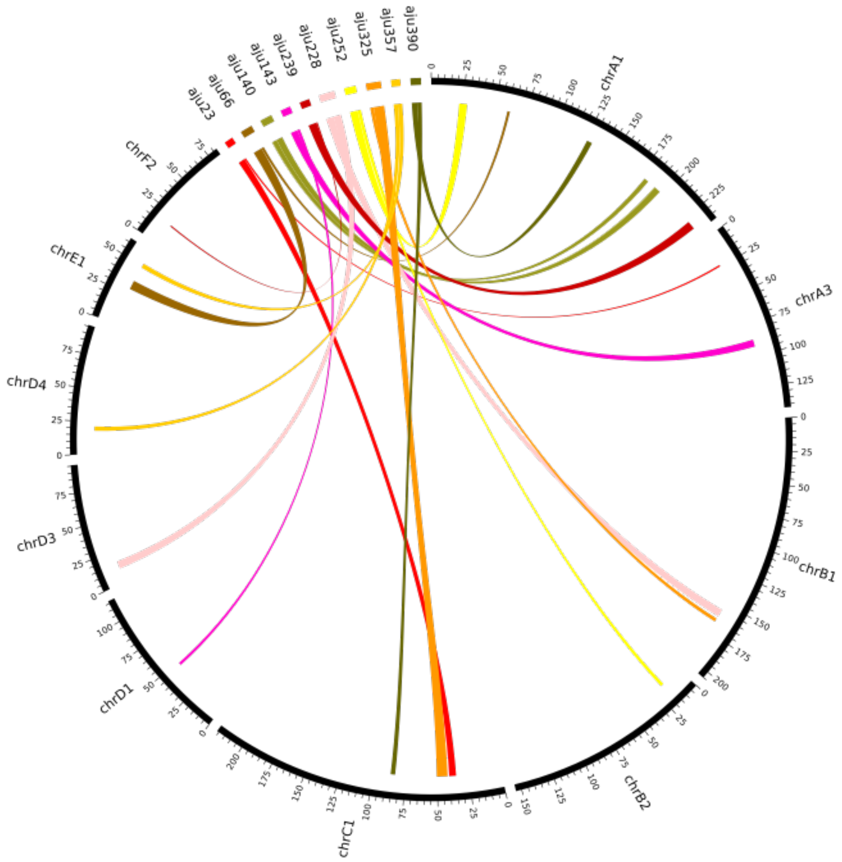

Figure S6: **Ten largest rearrangements between cat and cheetah genomes.** The rearrangements are shown between cat chromosomes and cheetah scaffolds.

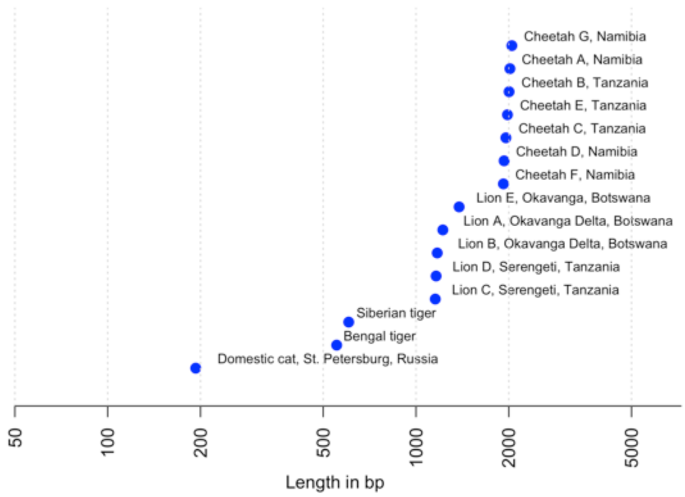

Figure S7: **Size of homozygosity stretches in Felidae genomes.** For each individual, the median length of homozygosity regions in its genome is shown.

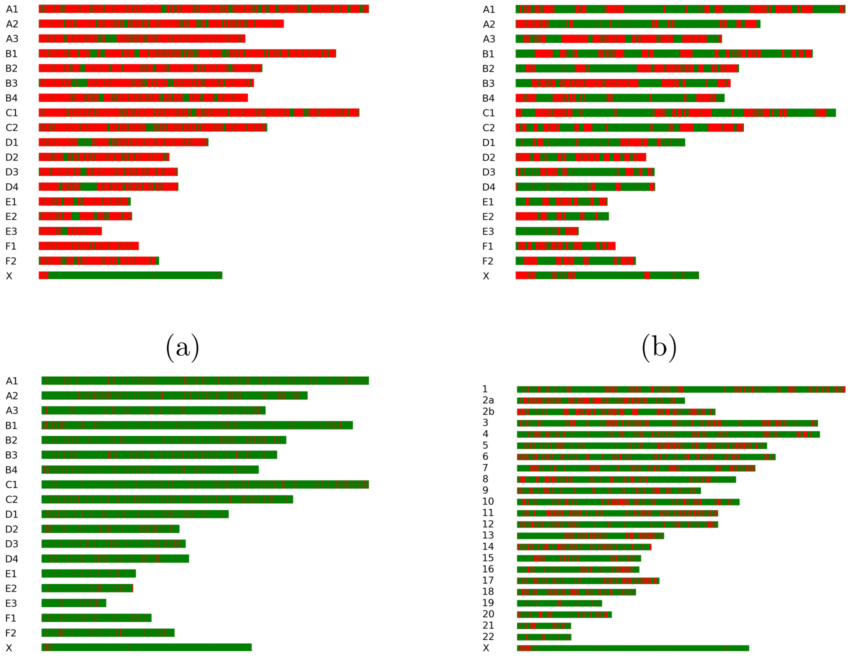

(a) (b) (c) (d)

Figure S8: **Ideograms of homozygosity regions in genomes of cat (Boris and Cinnamon), cheetah (Chewbacca) and mountain gorilla (Imfura).** The method used to obtain the figures is described in the legend of Figure 1d. (a) The ideogram of homozygosity regions in the genome of Boris — an outbred domestic cat from St. Petersburg [2]. Homozygous windows constitute 24.80% of the genome. (b) The ideogram of homozygous windows in the genome of Cinnamon — an inbred Abyssinian domestic cat [3, 2]. Homozygous windows constitute 62.63% of the genome. (c) The ideogram of homozygosity regions in the cheetah genome (Chewbacca). Homozygous windows constitute 93.20% of the genome. (d) The ideogram of homozygosity regions in the mountain gorilla genome (Imfura) [4]. Homozygous windows constitute 78.12% of the genome.

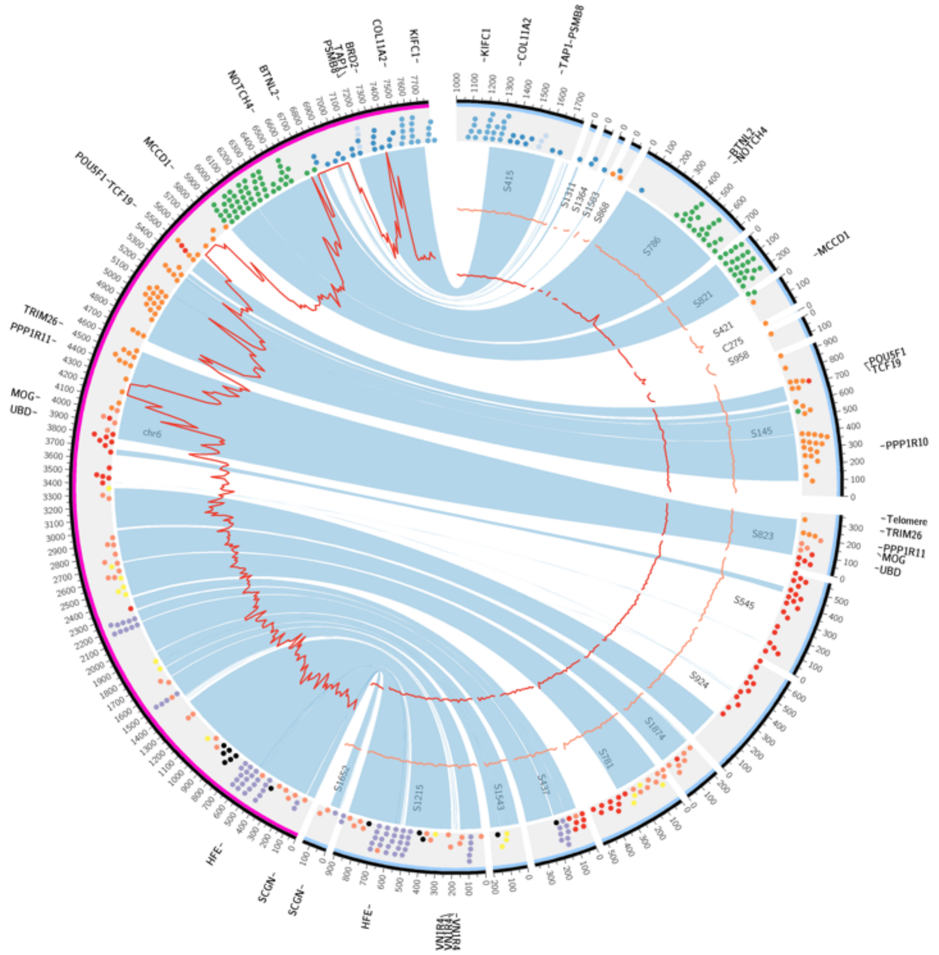

Figure S9: **Comparison of cheetah and human MHC regions.** Human is on the right side and cheetah on the left. SNV density for human (red) displayed with graphs inside the Circos plot. For two cheetahs populations SNV density displayed with orange (Tanzania) and red (Namibia) graphs.

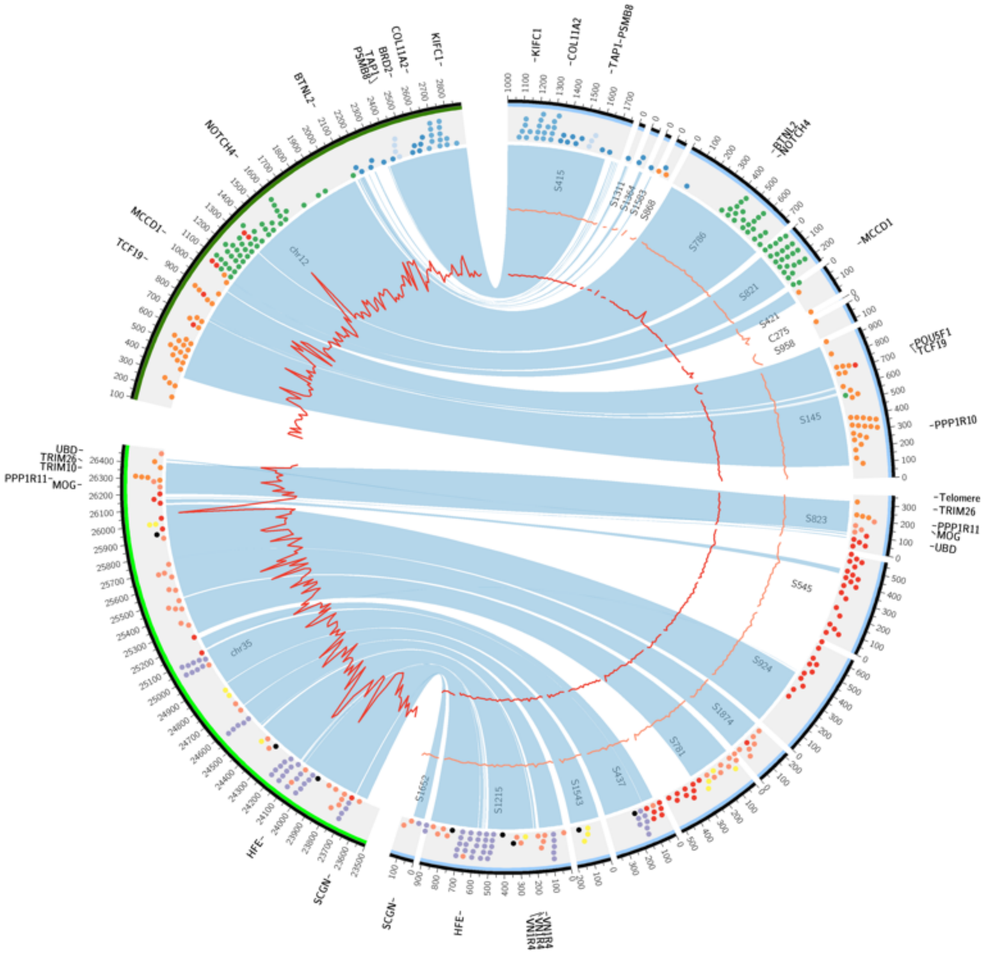

Figure S10: **Comparison of cheetah and dog MHC regions.** Dog is on the right side and cheetah on the left. SNV density for dog (red) displayed with graphs inside the Circos plot. For two cheetahs populations SNV density displayed with orange (Tanzania) and red (Namibia) graphs.

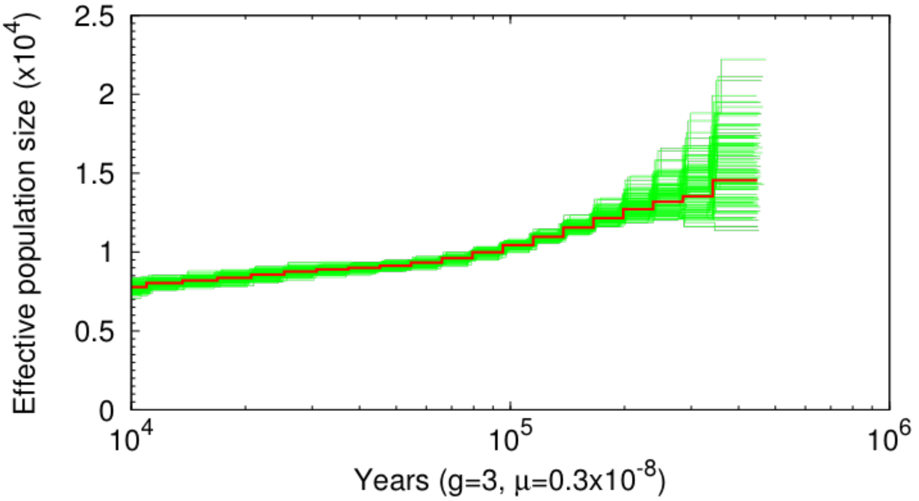

Figure S11: **Inferred historical population sizes by pairwise sequential Markovian coalescent analysis.** The x-axis gives time measured by pairwise sequence divergence and the y-axis gives the effective population size measured by the scaled mutation rate μ.

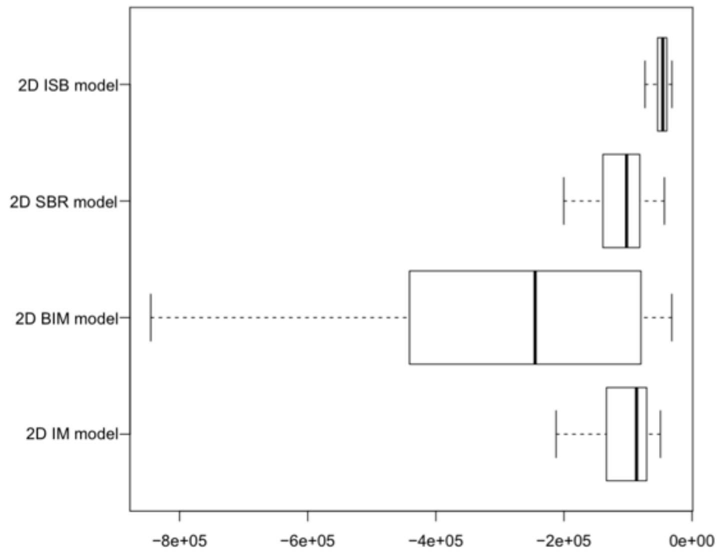

Figure S12: **Bootstrap values for DaDi demographic models.** We perform a total of 100 bootstraps to estimate the variance of the log-likelihood by randomly selecting 1 M of cheetah SNVs. Each one were performed four times, using IM, BIM, SBR and ISB models, respectively. The last model (designated as ISB) has the smallest variance and thus is considered the optimal model for cheetah.

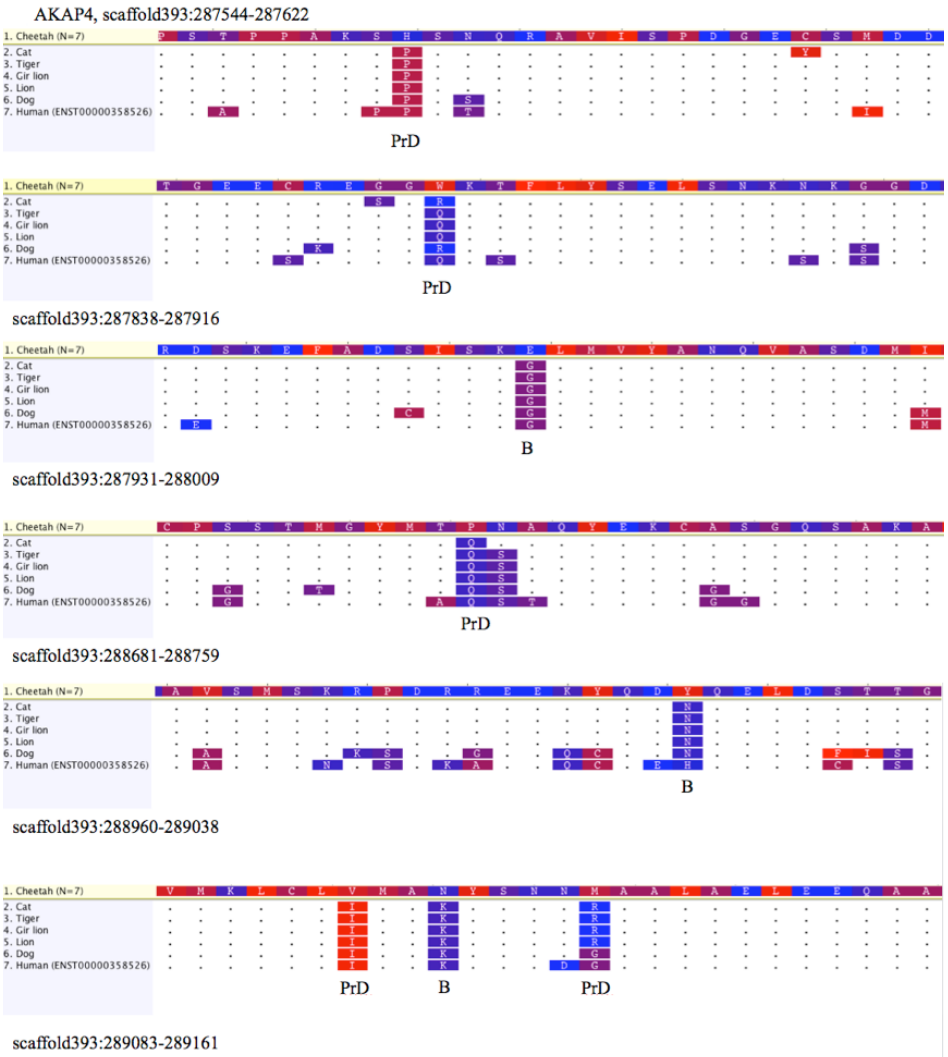

Figure S13: **Alignments of the *AKAP4* gene showing excess of possibly damaging mutations and large deletions according to the Polyphen2 database.** Amino-acid changes in proteins of other species are shown relative to the cheetah protein. The following variant effect notations are used: B — benign, PsD — possibly damaging and PrD — probably damaging.

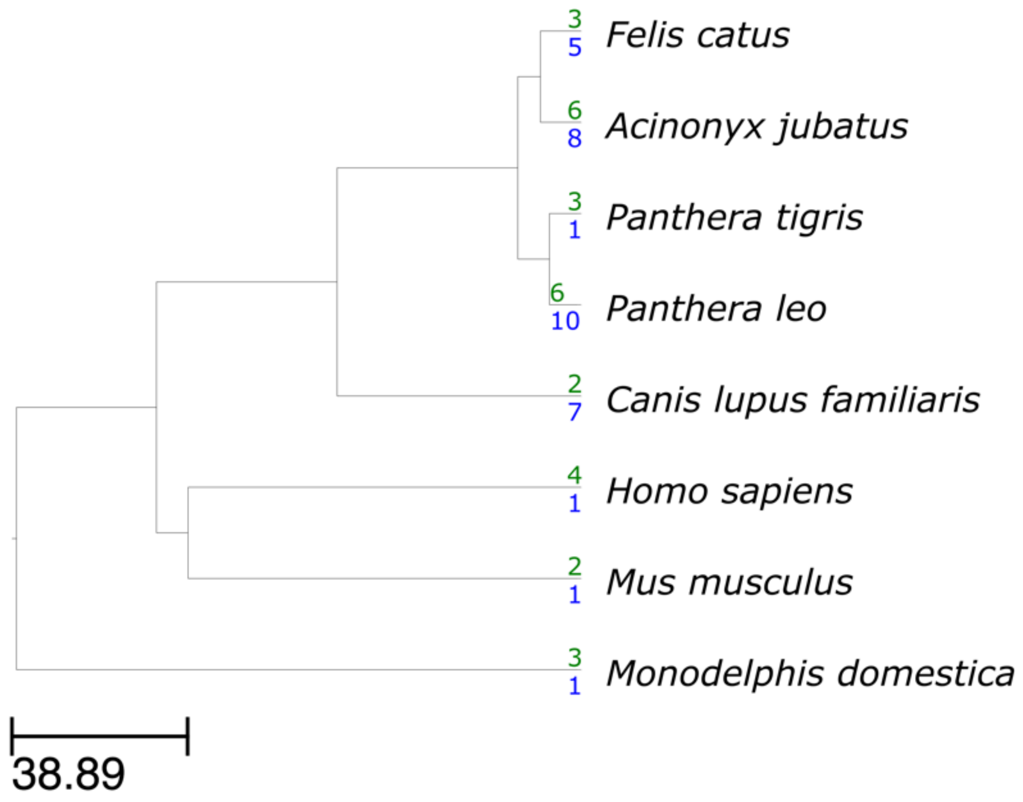

Figure S14: **Evolutionary history of LDHA/LDHC and LDHB gene families in mammals.** The history was reconstructed with CAFE 3.0 [5] using orthologous gene clusters (Figure 5b).

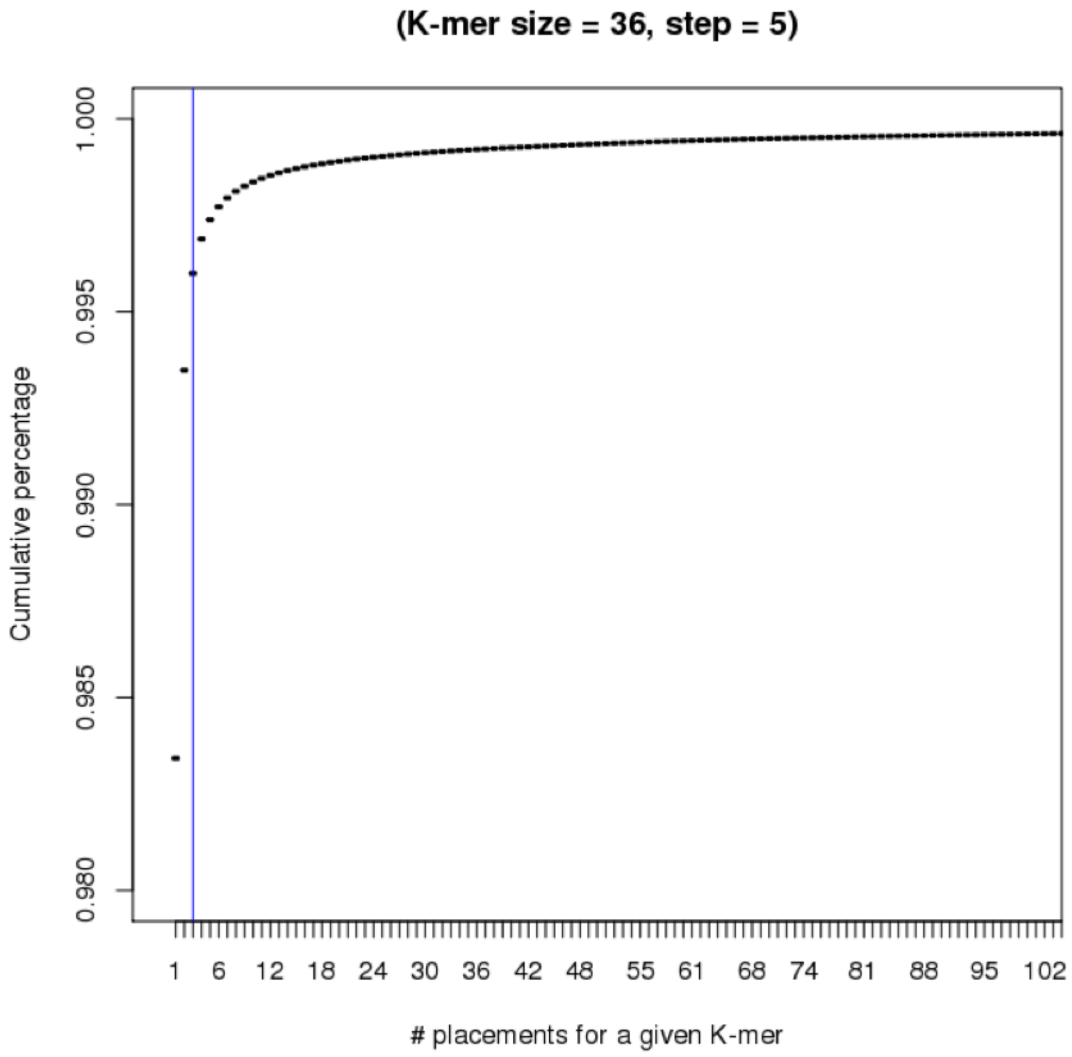

Figure S15: **36-mers cumulative distribution.**

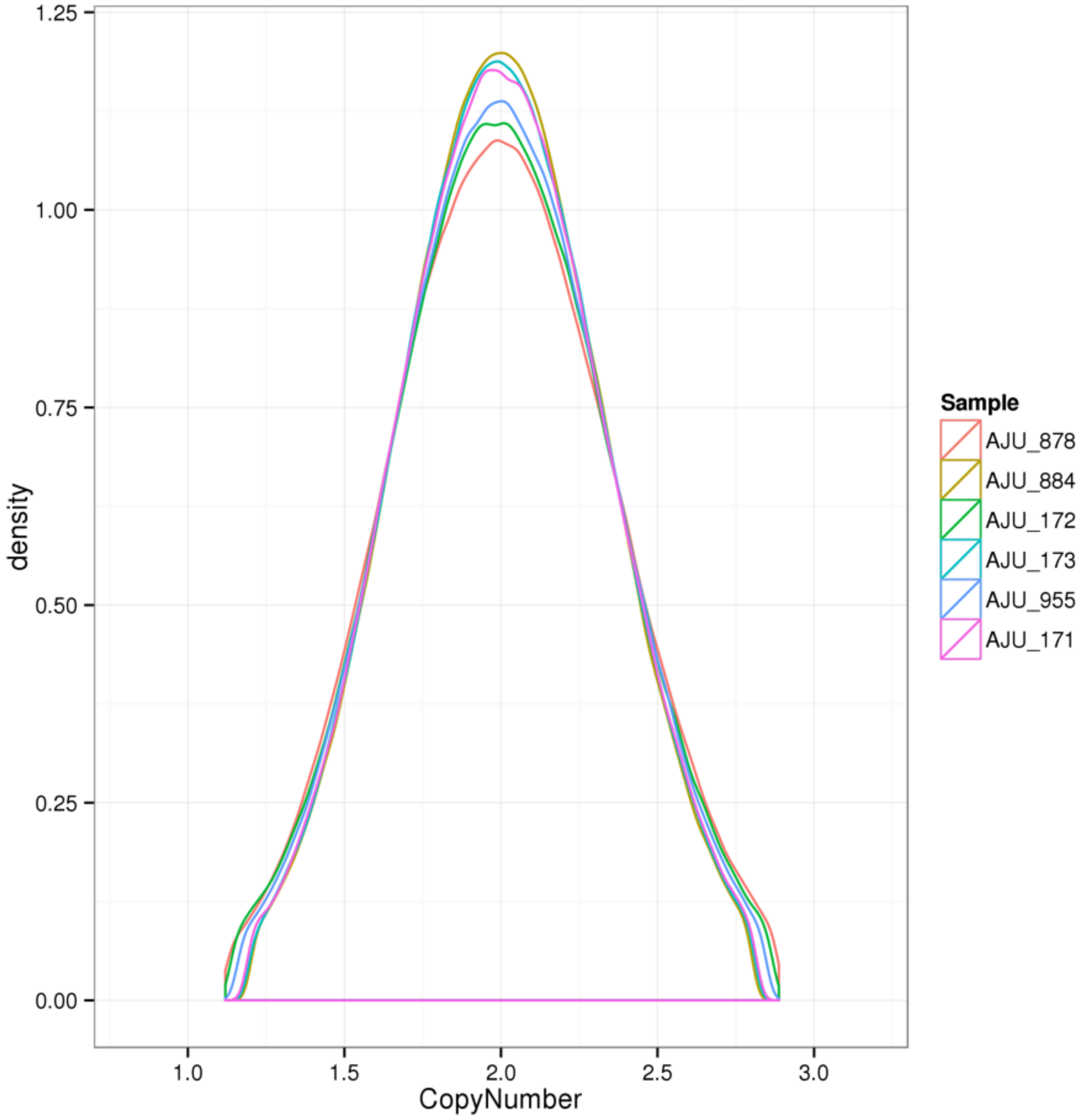

Figure S16: **Copy-number distribution in control regions.**

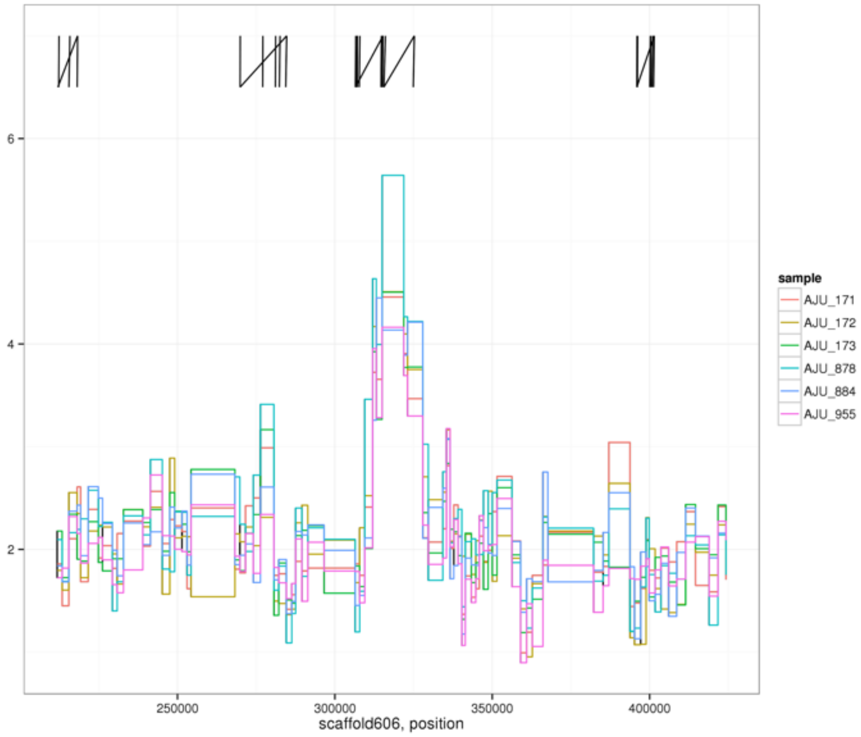

Figure S17: **Example of fixed duplications on scaffold606.**

References

[1] Liu B, Shi Y, Yuan J, Hu X, Zhang H, Li N, et al. Estimation of genomic characteristics by analyzing k-mer frequency in de novo genome projects. arXiv preprint arXiv:13082012. 2013;1308.

[2] Tamazian G, Simonov S, Dobrynin P, Makunin A, Logachev A, Komissarov A, et al. Annotated features of domestic cat–*Felis catus* genome. GigaScience. 2014;3(1):13.

[3] Pontius JU, Mullikin JC, Smith DR, Lindblad-Toh K, Gnerre S, Clamp M, et al. Initial sequence and comparative analysis of the cat genome. Genome Research. 2007;17(11):1675–1689.

[4] Xue Y, Prado-Martinez J, Sudmant PH, Narasimhan V, Ayub Q, Szpak M, et al. Mountain gorilla genomes reveal the impact of long-term population decline and inbreeding. Science. 2015;348(6231):242–245.

[5] Han MV, Thomas GW, Lugo-Martinez J, Hahn MW. Estimating gene gain and loss rates in the presence of error in genome assembly and annotation using CAFE 3. Molecular Biology and Evolution. 2013;30(8):1987–1997.
