# Supplementary material for: Genomic legacy of the African cheetah, Acinonyx jubatus
Source: Genome Biol. 2015 Dec 10;16:277. doi: 10.1186/s13059-015-0837-4 (PMC4676127; doi:10.1186/s13059-015-0837-4)
Supplement: Additional file 2 — Supplemental tables. Table S1. Sequenced cheetah reads for de novo genome assembly. Table S2. Re-sequenced cheetah reads for population analyses. Table S3. Estimated cheetah genome size. Table S4. Cheetah genome assembly information. Table S5. Reference-assisted assembly of cheetah chromosomes. Table S6. RepeatMasker results for transposable elements in carnivore genomes. Table S7. Total length of repeat regions in cheetah. Table S8. Tandem repeats in five carnivore genomes. Table S9. Complex tandem repeat families. Table S10. Protein-coding gene annotation. Table S11. Non-coding RNA annotation. Table S12. Nuclear mitochondrial genes. Table S13. Lengths of cheetah synteny blocks. Table S14. Cheetah rearrangements. Table S15. Called SNV statistics. Table S16. SNV effects by impact. Table S17. SNV effects by functional class. Table S18. SNV effects by genomic region. Table S19. SNV locations relative to genes. Table S20. SNV distribution in cheetah genome. Table S21. SNV distribution in tiger genomes. Table S22. SNV locations and effects in coding genes of Felidae genomes. Table S23. SNV counts in genes in domestic cat and tigers. Table S24. SNV counts in genes in cheetahs. Table S25. Nucleotide diversity in mitochondrial genomes of mammals. Table S26. Nucleotide diversity in MHC class I and II genes. Table S27. Demographic models and their log-likelihood values. Table S28. Population data by DaDi. Table S29. Reproductive system genes with identified function. Table S30. Filtration of cheetah reproduction system genes. Table S31. Nucleotide diversity of masked assemblies. Table S32. Statistics on autosomal segmental duplications. (PDF 127 kb) [file 13059_2015_837_MOESM2_ESM.pdf]

Supplemental Tables
for
Genomic Legacy of the African Cheetah,
Acinonyx jubatus

List of Tables

Table S1: Sequenced cheetah reads for de novo genome assembly 3
Table S2: Re-sequenced cheetah reads for population analyses . . 3
Table S3: Estimated cheetah genome size 3
Table S4: Cheetah genome assembly information 4
Table S5: Reference-assisted assembly of cheetah chromosomes . 5
Table S6: RepeatMasker results for transposable elements in carnivore genomes 6
Table S7: Total length of repeat regions in cheetah 6
Table S8: Tandem repeats in five carnivore genomes 6
Table S9: Complex tandem repeat families 7
Table S10: Protein-coding gene annotation 7
Table S11: Non-coding RNA annotation 7
Table S12: Nuclear mitochonrial genes 8
Table S13: Lengths of cheetah synteny blocks 8
Table S14: Cheetah rearrangements 9
Table S15: Called SNV statistics 9
Table S16: SNV effects by impact 9
Table S17: SNV effects by functional class 10
Table S18: SNV effects by genomic region 10
Table S19: SNV locations relative to genes 10
Table S20: SNV distribution in cheetah genome 11
Table S21: SNV distribution in tiger genomes 11
Table S22: SNV locations and effects in coding genes of Felidae genomes 12
Table S23: SNV counts in genes in domestic cat and tigers 12
Table S24: SNV counts in genes in cheetahs 13
Table S25: Nucleotide diversity in mitochondrial genomes of mammals 13
Table S26: Nucleotide diversity in MHC class I and II genes 14
Table S27: Demographic models and their log-likelihood values . . 15
Table S28: Population data by DaDi 15
Table S29: Reproductive system genes with identified function . . 16
Table S30: Filtration of cheetah reproduction system genes 17
Table S31: Nucleotide diversity of masked assemblies 18
Table S32: Statistics on automosomal segmental duplications . . . 19

Table S1: **The sequenced cheetah reads for the de novo assembly.** High quality data represents the clean data, which filter the low-quality score, duplication and PCR redundancy reads.

Insert size (bp)	Read length (bp)	Raw data (Gb)	High-quality data (Gb)	Sequence coverage (×)
170	100	102.20	67.97	28.56
500	100	50.48	38.70	16.26
800	100	38.49	26.40	11.09
2,000	49	33.89	18.53	7.79
5,000	49	45.71	10.44	4.39
10,000	49	27.27	6.51	2.73
20,000	49	24.26	9.76	4.10

Table S2: **The re-sequenced cheetah reads for the population analyses.** High quality data represents the clean data, which filter the low-quality score, duplication and PCR redundancy reads.

Insert size (bp)	Read length (bp)	Raw data (Gb)	High-quality data (Gb)	Sequence depth (×)	Total mapped bases (Gb)	% Reads after filtration
500	100	13.97	13.06	5.49	12.77	97.76
500	100	17.79	16.44	6.91	16.04	97.57
500	100	19.42	17.80	7.48	17.37	97.57
500	100	16.65	15.39	6.47	14.93	96.98
500	100	15.60	14.52	6.10	14.13	97.32
500	100	18.89	17.25	7.25	16.76	97.14

Table S3: **The estimated genome size for cheetah.** Using more than 25× raw reads to estimate the cheetah genome size.

Kmer size	# Kmers	Kmer depth (×)	Estimated genome size (bp)	Used bases (bp)	Depth (×)
17	50,303,647,592	21	2,395,411,790	68,595,883,080	28.64

Table S4: **The assembly information.** The total genome size of assembly for cheetah is about 2.38 Gb.

	Contig		Scaffold	
	Size (bp)	Number	Size (bp)	Number
N90	6,675	87,953	817,533	784
N80	11,732	61,999	1,417,882	568
N70	16,749	45,401	1,986,090	427
N60	22,136	33,294	2,460,097	320
N50	28,224	23,952	3,121,442	233
Longest	304,265		13,046,067	
Total size (bp)	2,333,696,654		2,375,786,546	

Table S5: **Statistics on the reference-assisted assembly of cheetah chromosomes.** The intragaps column gives the total length of gaps within the scaffolds that were assembled to a chromosome. The intergaps column gives the total length of gaps situated between the scaffolds of a chromosome.

Chromosome	# Intragaps	# Intergaps	# Scaffolds	Total length (bp)
A1	2,676,546	21,786,027	95	244,616,204
A2	3,095,843	3,421,688	94	169,488,250
A3	2,234,714	6,462,378	72	146,614,859
B1	2,396,843	15,769,704	111	208,967,989
B2	2,142,340	5,807,146	94	158,072,724
B3	2,425,085	4,951,280	82	153,641,819
B4	2,111,389	8,753,595	58	145,667,879
C1	3,110,905	12,616,103	114	225,903,316
C2	1,830,801	4,896,233	79	161,030,742
D1	2,203,422	7,353,918	80	119,018,581
D2	1,168,337	4,642,075	42	90,695,764
D3	1,432,032	3,536,984	68	98,158,025
D4	1,825,900	4,074,642	61	98,516,449
E1	1,707,661	8,039,036	65	64,109,970
E2	1,469,428	6,987,499	60	65,905,433
E3	1,264,052	3,897,881	21	43,588,344
F1	1,453,485	2,873,379	44	72,411,080
F2	1,193,297	2,303,763	46	84,041,130
X	4,587,499	10,723,712	233	131,400,970
Total	40,329,579	138,897,043	1,519	2,481,849,528
Total length of assembled fragments without intergaps:				2,342,952,485
Total length of all fragments:				2,375,874,546
Total length of unplaced and unlocalized fragments:				32,922,061

Table S6: **RepeatMasker results for TE in five carnivore genomes.** For each genome, percentages of total length of identified repeats relative to the genome size are given. All five genomes have similar percentage of masked genomes and similar proportion between the repeat families.

	Cheetah	Lion	Cat	Tiger	Dog
Masked portion	39.48	38.94	39.17	39.15	39.97
SINEs	10.80	10.82	10.98	10.84	10.37
LINEs	20.28	19.85	20.02	19.95	20.18
LTR elements	5.27	5.19	5.08	5.23	4.94
DNA elements	2.94	2.89	2.82	2.93	2.79
Other	0.14	0.14	0.22	0.15	1.64
Unclassified	0.05	0.05	0.05	0.05	0.05
Small RNA	8.03	8.10	8.32	8.09	7.81

Table S7: **The total length of repeat regions in cheetah.** In total, there are about 869 Mb (36.58% of the genome) of repeat regions, including DNA, LINE, LTR, SINE and other unclassified types.

TE class	Size (bp)	Percentage of genome (%)
DNA	77,243,347	3.25
LINE	510,499,028	21.49
LTR	121,299,829	5.11
SINE	241,513,753	10.17
Other	5,697	0.00
Unknown	7,546,303	0.32
Total	868,991,259	36.58

Table S8: **Tandem repeats in five carnivore genomes.** Felidae genomes have similar TRs profile and differ from the dog genome TR profile.

	Cheetah	Lion	Tiger	Cat	Dog
All TRs	851,094	955,312	891,574	882,953	1,085,732
Microsatellites	329,761	366,015	339,997	361,992	309,032
Perfect microsatellites	132,548	150,769	135,625	152,612	135,903
Complex TRs	3,457	3,555	3,270	4,038	7,492
Large TRs >1 kbp	1,284	2,113	1,727	2,077	4,733
Large TRs >3 kbp	165	263	196	245	2,931
Large TRs >10 kbp	11	8	6	7	715

Table S9: **Complex tandem repeat families.**

Family	# Arrays	Monomer length in bp	GC%	Predicted features
Ajub33A	177	33	51.0	Predicted pericentromeric
Ajub483A	65	483	50.5	Predicted pericentromeric/pretelomeric
Ajub25A	50	25	65.0	—
Ajub113A	47	113	39.0	Predicted pericentromeric
Ajub84A	277	84	40.0	Zinc-finger domains

Table S10: **Statistics on protein-coding gene annotation.** In total, more than 20,000 protein-coding genes were predicted.

	Number	Total length (Mb)	Percentage of genome (%)
Genes	20,343	601.20	25.30
CDS	174,408	29.00	1.22

Table S11: **Statistics on non-coding RNA annotation.** For annotation of non-coding RNA elements, we used tRNAscan-SE (version 1.23) to search for tRNA-coding sequences. Vertebrate rRNA from the European ribosomal RNA database was used to predict DBM rRNA sequences. Rfam (version 9.1) combined with Infernal were used to predict small nuclear RNAs (snRNAs) and microRNAs (miRNAs).

Type	Subtype	Copy Number	Average length (bp)	Total length (bp)	% of genome
miRNA		43,878	102	4,457,926	0.187633
tRNA		62,321	84	5,230,323	0.220143
rRNA	rRNA	530	81	43,123	0.001815
	18S	27	176	4,759	0.000200
	28S	96	100	9,578	0.000403
	5.8S	1	65	65	0.000003
	5S	406	71	28,721	0.001209
snRNA	snRNA	1,605	118	189,141	0.007961
	CD-box	312	94	29,247	0.001231
	HACA-box	241	139	33,446	0.001408
	Splicing	1,014	120	122,032	0.005136

Table S12: **Partial and complete nuclear mitochondrial genes.** In total, 143 potential Numts were found in the cheetah genome, their total size is 105,629 bp. From those, 50 are complete genes and 93 are partial matches.

Gene name	# Gene copies	Gene name	# Gene copies
Complete		Partial	
RNA	10	*RNA*	31
ND6	7	*COX1*	12
ND1	5	*CYTB*	9
ND3	5	*COX2*	7
COX3	4	*ATP6*	7
ND4L	4	*ND1*	7
ATP8	3	*ND5*	6
CYTB	3	*ND2*	5
ND2	3	*COX3*	4
COX2	2	*ND4*	3
ATP6	2	*ATP8*	1
COX1	1	*ND3*	1
ND4	1		

Table S13: **Lengths of synteny blocks in cheetah in relation to other species based on 5 species alignment.**

Synteny block length statistics	*Felis catus*	*Panthera tigris*	*Panthera leo*	*Canis familiaris*
Min	0.3 Mb	0.3 Mb	0.3 Mb	0.3 Mb
Max	13.0 Mb	10.9 Mb	8.7 Mb	13.0 Mb
Median	1.4 Mb	1.2 Mb	0.9 Mb	1.4 Mb
Total length	2.34 Gb	2.31 Gb	2.27 Gb	2.28 Gb
Fraction of cheetah genome	98.6%	97.1%	95.6%	96.0%
Median density of aligned anchors in cheetah synteny blocks	96.7%	96.0%	95.3%	87.1%

Table S14: **The number of cheetah rearrangements obtained from 5 species multiple genome alignments with tiger, cat and dog genomes.** In parentheses, the numbers of rearrangements are given after excluding the whole-scaffold ones.

Pairwise alignment	*Panthera tigris*	*Felis catus*	*Panthera leo*	*Canis familiaris*
Reversal	0	7 (1)	4 (1)	14 (1)
Fusion	781 (22)	1,067 (11)	217 (3)	1,034 (178)
Fission	163 (31)	4 (3)	92 (4)	0
Translocation	188 (27)	39 (4)	1,080 (113)	87 (19)
Overall	1,132 (80)	1,117 (19)	1,393 (121)	1,135 (198)

Table S15: **Statistic on called SNVs.** Total SNV count includes all SNV in coding regions and in repetitive elements. Singletons are SNV encountered only in one sample. An available sites is a genome size without unknown base pairs (Ns). SNV rate calculated as the total SNV number divided by the number of available sites.

Total SNVs	# Singleton	Available sites	SNVs rate
3,438,824	1,432,827	2,048,690,294	0.0016

Table S16: **SNV effects grouped by impact.** High, Low, Moderate and Modifier effects relate to potential impact on gene function, for more description please check the snpEff manual (`http://snpeff.sourceforge.net/SnpEff_manual.html`). AJU_NAM and AJU_TAN stand for cheetahs (*A. jubatus*) from Namibia and Tanzania, respectively.

	Modifier	Moderate	Low	High
AJU_NAM_1	526,197	4,029	2,853	647
AJU_TAN_1	499,790	3,701	2,682	629
AJU_TAN_2	534,540	3,958	2,827	813
AJU_NAM_2	533,605	4,010	2,868	828
AJU_TAN_3	496,620	3,779	2,709	617
AJU_NAM_3	531,055	3,889	2,942	799
AJU_NAM_4	526,189	4,882	2,939	680

Table S17: **SNV effects grouped by functional class.** Missense and nonsense effects result in an amino acid change of the protein encoded by a gene. A missense SNV leads to substitution of a single amino acid in a protein. A nonsense SNV causes a premature stop codon. A silent SNV does not alter the protein translated from a gene. AJU_NAM and AJU_TAN stand for cheetahs (*A. jubatus*) from Namibia and Tanzania, respectively.

	Silent	Missense	Nonsense
AJU_NAM_1	2,322	4,041	134
AJU_TAN_1	2,144	3,720	109
AJU_TAN_2	2,206	3,971	159
AJU_NAM_2	2,285	4,032	134
AJU_TAN_3	2,174	3,797	106
AJU_NAM_3	2,361	3,909	132
AJU_NAM_4	2,321	4,901	130

Table S18: **SNV effects grouped by genomic region.** Both regions inside and outside protein-coding genes were considered. Upstream and downstream regions were defined as 5 kbp stretches surrounding a gene. Putative splice sites were considered to be of 2 bp long. AJU_NAM and AJU_TAN stand for cheetahs (*A. jubatus*) from Namibia and Tanzania, respectively.

	Downstream	Exon	Intergenic	Intron	Splice site			Upstream
					Acceptor	Donor	Region	
AJU_NAM_1	20,906	6,761	364,130	122,791	58	179	531	18,370
AJU_TAN_1	19,804	6,243	347,735	114,904	50	181	538	17,347
AJU_TAN_2	21,203	6,735	370,760	123,953	70	173	620	18,624
AJU_NAM_2	21,253	6,879	370,573	122,575	49	194	584	19,204
AJU_TAN_3	19,661	6,350	343,955	115,369	49	172	534	17,635
AJU_NAM_3	20,971	6,798	369,291	122,072	54	198	580	18,721
AJU_NAM_4	21,128	7,625	363,638	122,511	59	199	618	18,912

Table S19: **SNVs locations in the cheetah genome relative to protein-coding genes.**

SNV location	Number
Outside genes	1,342,346
Within genes	478,073
Within gene exons	24,343
Within gene introns	453,730

Table S20: **Heterozygous SNV distribution in cheetah genome.** For each sample, we considered SNVs that were heterozygous in it. To estimate SNV distribution in the genome, we split it to non-overlapping 50 kbp windows and calculated minimum, median, average and maximum SNV counts in them. Heterozygous SNV rate was calculated as ratio of the heterozygous SNV number and the genome length. AJU_NAM and AJU_TAN stand for cheetahs (*A. jubatus*) from Namibia and Tanzania, respectively.

Sample	# Heterozygous SNVs						Heterozygous
	Min	Median	Average	Max	In windows	Total	SNV rate
AJU_NAM_1	0	9	10.35	248	484,403	494,308	0.00020
AJU_TAN_1	0	8	9.82	330	459,588	469,343	0.00019
AJU_TAN_2	0	9	10.51	337	491,758	501,858	0.00021
AJU_NAM_2	0	9	10.49	253	490,857	500,450	0.00021
AJU_TAN_3	0	8	9.75	343	456,263	466,218	0.00019
AJU_NAM_3	0	9	10.44	348	488,607	498,729	0.00020
AJU_NAM_4	0	9	10.37	335	484,951	494,299	0.00020

Table S21: **Heterozygous SNV distribution in tiger genomes.** The SNV distribution was estimated in the same way as in Table S20. PTI_BEN and PTI_SIB stand for Bengal (*P. tigris tigris*) and Siberian (*P. tigris altaica*) tigers, respectively.

Sample	# Heterozygous SNVs						Heterozygous
	Min	Median	Average	Max	In windows	Total	SNV rate
PTI_BEN	0	16	20.38	552	970,056	980,686	0.0004
PTI_SIB	0	6	15.19	521	722,882	732,809	0.0003

Table S22: **Location and effect of heterozygous SNVs in coding genes of Felidae genomes.** FCA, PTI_BEN and PTI_SIB stand for domestic cat (*F. catus* "Boris"), Bengal (*P. tigris tigris*) and Siberian (*P. tigris altaica*) tigers, respectively. AJU_NAM and AJU_TAN stand for cheetahs (*A. jubatus*) from Namibia and Tanzania, respectively.

	SNV effect		Location in gene	
	Synonymous	Non-synonymous	Exon	Intron
FCA	20,713	12,143	34,002	947,752
AJU_NAM_1	2,321	4,026	6,755	122,547
AJU_TAN_1	2,144	3,701	6,235	114,652
AJU_TAN_2	2,205	3,953	6,690	123,621
AJU_NAM_2	2,284	4,006	6,832	122,307
AJU_TAN_3	2,172	3,776	6,333	115,103
AJU_NAM_3	2,360	3,888	6,759	121,818
AJU_NAM_4	2,320	4,879	7,611	122,242
PTI_BEN	5,394	4,197	9,677	293,605
PTI_SIB	5,008	9,230	14,450	217,882

Table S23: **The numbers of genes with different heterozygous SNV counts in their coding regions for domestic cat (*F. catus*, FCA), Bengal (*P. tigris tigris*, PTI_BEN) and Siberian (*P. tigris altaica*, PTI_SIB) tigers.**

#SNVs in gene	FCA	PTI_BEN	PTI_SIB
0	11,737	15,256	13,261
1	3,625	2,943	3,742
2	2,171	1,085	1,564
3	1,381	449	734
4	857	170	392
5	587	103	195
6	393	64	146
7	291	46	68
8	167	31	40
9	159	19	12
>10	522	60	72

Table S24: **The numbers of genes with different heterozygous SNV counts in their coding regions for cheetahs.** AJU_NAM and AJU_TAN stand for cheetahs (*A. jubatus)* from Namibia and Tanzania, respectively.

#SNVs in gene	AJU_NAM_1	AJU_TAN_1	AJU_TAN_2	AJU_NAM_2	AJU_TAN_3	AJU_NAM_3	AJU_NAM_4
0	16,026	16,334	16,187	16,073	16,268	16,101	15,441
1	3,070	2,891	2,942	3,009	2,946	3,018	3,425
2	790	701	777	813	711	769	938
3	238	226	219	238	217	241	324
4	91	86	89	109	86	105	96
5	48	30	49	22	43	35	44
6	27	20	30	26	18	19	23
7	19	10	10	8	11	17	14
8	6	9	7	6	10	7	7
9	2	11	3	6	5	4	9
>10	25	24	29	32	27	26	21

Table S25: **Nucleotide diversity in mitochondrial genomes among mammals.** Pi measure is defined as the average number of nucleotide differences per site between any two DNA sequences chosen randomly from the sample population. Standard deviation (SD) of Pi is also given.

Species	Pi	SD
A. jubatus (Tanzania)	8.00E-05	3.00E-05
A. jubatus (Namibia)	7.10E-04	3.30E-04
B. bison	7.90E-04	7.00E-05
B. taurus	8.60E-04	1.50E-04
O. orca	1.20E-03	2.10E-04
H. sapiens	1.65E-03	8.00E-05
C. lupus familiaris	3.31E-03	1.00E-04
E. caballus	3.80E-03	1.00E-04
C. lupus	5.18E-03	3.70E-04
P. troglodytes	5.38E-03	2.00E-07

Table S26: **Nucleotide diversity in predicted class II and I MHC genes.** Gene completeness was assessed by presence of conservative domains: if the domains were present partially, then a gene was denoted as of partial completeness, otherwise — as of full. S and NS stand for synonymous and non-synonymous SNVs. MHC gene annotation for cat (*F. catus*) was taken from [1]. The NA value for a cat MHC gene means that the gene was not annotated in the original paper.

Class	Gene name	Completeness	#SNVs in *A. jubatus*		#SNVs in *F. catus*	
			S	NS	S	NS
II	*DRBp*	Full	1	4	NA	NA
II	*DRAp*	Partial	0	3	NA	NA
II	*DNA*	Full	1	5	NA	NA
II	*DMA*	Full	0	2	NA	NA
II	*DMB*	Full	0	0	NA	NA
II	*DOB*	Full	1	0	NA	NA
II	*DRB4*	Full	1	1	8	25
II	*DRB1*	Partial	0	0	1	2
II	*DRA1*	Full	0	0	2	0
II	*DRB3*	Full	0	0	5	13
II	*DRA2*	Full	0	0	0	0
II	*DRA3*	Full	0	0	0	0
I	*FLA-A*	Full	1	2	1	0
I	*FLA-C*	Partial	0	2	2	1
I	*FLA-E*	Partial	0	0	0	0
I	*FLA-F*	Partial	0	1	4	40
I	*FLA-H*	Partial	0	0	15	50
I	*FLA-J*	Partial	0	0	6	7
I	*FLA-K*	Full	0	0	0	1
I	*FLA-L*	Partial	0	3	1	1
I	*FLA-O*	Full	0	0	3	4
I	*FLA-Q*	Partial	0	2	NA	NA

Table S27: **Tested demographic models and their optimized log-likelihood values.** The last model has the largest likelihood. We also perform a total of 100 bootstraps to estimate the variance of the result by randomly selecting the real data. We found the variance of likelihood in the last model is much smaller compared with other models (see Figure 3 legend).

Model	Scenario	Optimized log-likelihood
1	2D IM model (ancestral population splits into two subpopulations accompanying with migration from each other.)	-51,979.09
2	2D BIM model (the ancestral population undergoing a bottleneck, and then splits)	-77,657.60
3	2D SBR model (population bottleneck followed by population isolation and then population recovering)	-50,753.12
4	2D ISB model (population isolation after a long time increasing of ancestor population and the isolated populations experience bottleneck respectively)	-43,587.07

Table S28: **Summary of real population data calculated from the DaDi result as per Model 4 — 2D ISB (see Figure 3a).**

	Bounds	
	Lower	Upper
Migration rate from the east subpopulation to the west subpopulation	0.000994	0.001095
Migration rate from the west subpopulation to the east subpopulation	0.001049	0.001166
The population size before split	134,873	150,096
Total size of the east subpopulation after split	35,428	39,959
Total size of the west subpopulation after split	119,416	133,272
Isolated east subpopulation size after bottleneck	111	124
Isolated west subpopulation size after bottleneck	111	126
Population increasing time before split in years	100,089	113,518
Population split time in years	11,084	12,589
Subpopulations bottleneck time in years	11,208	12,728

Table S29: **Short list of reproductive system genes with identified function.** Severity of mutations was assessed using the Polyphen2 database [2].

Ensembl transcript ID	Gene name	P-value	Effect description
ENST00000233078	*DAZ1*	1.73E-20	Whole-gene deletion causes azoospermia in human. Cheetah has 1 putative deletion and 2 possibly damaging mutations.
ENST00000295228	*INHBB*	3.58E-07	2 probably damaging mutations.
ENST00000377847	*PAX5*	1.35E-06	1 probably damaging mutation.
ENST00000368457	*PYGO2*	1.49E-06	Several high-confidence possibly damaging SNVs in cheetah: residues 96, 97 and 105 altered in cheetah but highly conserved in other species.
ENST00000374273	*SPAG4*	4.62E-04	Potential misalignment.
ENST00000399635	*TSSK2*	2.71E-03	The gene plays an important role in spermatogenesis and is associated with infertility in human. There are 3 cheetah-specific mutations with moderate effect, nonconserved for other Felidae species.
ENST00000396368	*KIA0430*	2.84E-03	The important oogenesis-related gene with several putatively damaging mutations in cheetah.
ENST00000394337	*CCDC135*	3.46E-03	The important non-conserved gene containing several cheetah-specific mutations.
ENST00000585580	*TSSK6*	6.06E-03	Two cheetah-specific mutations in a conserved region which were identified by PolyPhen2 as benign; also a deletion was located in an interesting region.
ENST00000225538	*P2RX1*	6.08E-03	3 cheetah-specific mutations, one of them is benign but is situated after the conserved Cysteine residue.
ENST00000356545	*RSPO1*	1.33E-02	Potential misalignment.
ENST00000358526	*AKAP4*	1.58E-02	The gene of proved importance for sperm motility. Cheetah has 5 specific missense mutations; 3 of them are probably damaging.
ENST00000252677	*BMP15*	1.62E-02	The gene plays an important role in ovarian development; cheetah has 1 possibly damaging mutation.
ENST00000266991	*DHH*	2.13E-02	The gene is associated with gonadal disgenesis; cheetah has 1 probably damaging mutation.
ENST00000274008	*SPATA5*	2.31E-02	1 probably damaging mutation
ENST00000341206	*LCN6*	2.77E-02	1 probably damaging mutation
ENST00000328739	*VMO1*	4.61E-02	Potential misalignment.
ENST00000395858	*MOV10L1*	4.88E-02	1 probably damaging mutation

Table S30: **Filtration steps applied to genes related to reproduction system of cheetah.**

Step	Description	# Genes
1	Full set of reproduction-related human genes according to Gene Ontology (GO term `GO:0000003`).	964
2	1:1 orthology relationship for cat, human, dog, cheetah and tiger.	656
3	Dn/Ds values are significantly higher ($p<0.05$ without multiple-test correction) in cheetah than other studied species.	92
4	Genes having direct link with studied disease phenotype according to open source data.	13

Table S31: **Nucleotide composition of differently masked assemblies.** The following repeat-masking tools were applied: RM — RepeatMasker [3], TRF — Tandem Repeat Finder [4], kmer — the k-mer based approach (Figure S15; Subsection of "Materials and Methods"). SL denotes filtration of scaffolds by their length; short scaffolds were removed.

Assembly	#Scaffolds	Nucleotide composition						%Masked
		#Total	A	T	C	G	N	
Non-masked	40,077	2,375,874,546	685,085,510	684,898,432	481,940,477	481,891,179	42,058,948	1.77
RM + TRF	40,077	2,375,874,546	380,646,313	380,798,506	275,810,184	275,746,994	1,062,872,550	44.74
RM + TRF + kmer	40,077	2,375,874,546	377,139,850	377,291,178	272,922,019	272,864,219	1,075,657,281	45.27
RM + TRF + kmer + SL	1,652	2,363,937,210	375,835,592	375,991,860	271,842,430	271,784,545	1,068,482,783	45.20

Table S32: **Overview of segmental duplications (SD) on autosomes and coverage of cheetah samples.** AJU_NAM and AJU_TAN stand for cheetahs (*A. jubatus*) from Namibia and Tanzania, respectively.

Sample	Number of autosomal SD	Bps in autosomal SD	Sample Coverage
AJU_NAM_1	520	4,744,441	4.23
AJU_TAN_1	490	4,492,923	5.39
AJU_TAN_2	460	4,354,394	5.89
AJU_NAM_2	523	4,883,730	5.05
AJU_TAN_3	568	5,430,887	4.73
AJU_NAM_3	486	4,542,034	5.73

References

[1] Yuhki N, Mullikin JC, Beck T, Stephens R, O'Brien SJ. Sequences, annotation and single nucleotide polymorphism of the major histocompatibility complex in the domestic cat. PLoS One. 2008;3(7):e2674.

[2] Adzhubei IA, Schmidt S, Peshkin L, Ramensky VE, Gerasimova A, Bork P, et al. A method and server for predicting damaging missense mutations. Nature Methods. 2010;7(4):248–249.

[3] Smit AF, Hubley R, Green P. RepeatMasker Open-3.0; 1996–2010.

[4] Benson G. Tandem repeats finder: a program to analyze DNA sequences. Nucleic Acids Research. 1999;27(2):573.
